# Processing-Induced Variations in Bamboo Leaf Powder: Effects of Fixation Methods on Color Stability, Volatile Compounds, and Sensory Profiles

**DOI:** 10.3390/foods14223898

**Published:** 2025-11-14

**Authors:** Qi Wang, Zhaojun Wang, Qiuming Chen, Maomao Zeng, Jie Chen, Benu Adhikari, Fengxian Guo, Zhiyong He

**Affiliations:** 1 State Key Laboratory of Food Science and Resources, Jiangnan University, Wuxi 214122, China; 2 School of Food Science and Technology, Jiangnan University, Wuxi 214122, China; 3 School of Science, RMIT University, Melbourne, VIC 3083, Australia; 4 College of Oceanology and Food Science, Quanzhou Normal University, Quanzhou 362000, China

**Keywords:** thermal processing, bamboo leaf powder, GC-MS/O, characteristic odors, dynamic changes

## Abstract

Fixation is a necessary step in bamboo leaf powder processing and plays a decisive role in determining its color, aroma, and taste. It is irreplaceable for maintaining quality, stability, and forming unique sensory characteristics. In this study, optimal conditions for steamed bamboo leaf powder (SBL), baked bamboo leaf powder (BBL), and blanched bamboo leaf powder (BCBL) were determined by measuring chlorophyll content, color parameters, and enzyme inactivation. In addition, volatile organic compounds (VOCs) in bamboo leaf powder processed with different fixation methods were analyzed using gas chromatography–mass spectrometry (GC-MS), gas chromatography–olfactometry (GC-O), and relative odor activity value (ROAV). Steaming for 120 s, baking for 60 s, and blanching for 30 s effectively preserved color, with a* values of −1.37, −1.44, and −1.62, all superior to untreated bamboo leaf powder (UBL). Among them, BCBL showed the best color stability, with the lowest color difference (ΔE = 0.66) compared with fresh bamboo leaves (FBLs). Results showed that BBL retained the highest VOC abundance (15.67% of FBLs), followed by SBL (5.73%) and BCBL (5.48%). Hexanal, nonanal, linalool, and α-ionone were identified as key aroma contributors, forming green, fresh, and floral notes. Sensory differences were evident: SBL exhibited strong seaweed-like and roasted notes, BCBL showed partial loss of characteristic aromas, while BBL preserved grass, fruity, and woody attributes. These findings highlight the significant influence of fixation methods on aroma-active compounds and color stability, providing a theoretical basis for producing bamboo leaf powder with superior sensory quality.

## 1. Introduction

Globally, bamboo forests cover approximately 35 million hectares, with Asia accounting for over 65% of this area. China, India, and Myanmar are the primary countries with significant bamboo distribution, and China alone accounts for one-third of the global total, ranking it first worldwide [1]. The country contributes over 40% of the global bamboo industry, with applications in construction materials, household products, and livestock feed. The bioactive profile of bamboo leaves includes flavonoids, polysaccharides, phenolic acids, amino acids, and lactones, all of which contribute to their antioxidant, anti-inflammatory, and free radical–neutralizing properties, as well as potential cardiovascular advantages [2]. Throughout history, many medical texts in China have documented the medicinal value and edible uses of bamboo leaves. Even today, bamboo leaves are still used for medicinal and culinary purposes in certain regions of China. In recent years, bamboo resource utilization has shifted toward high-value processing, enhancing its economic significance. Bamboo-derived products, including leaf extracts, bamboo vinegar, medicinal foods, and nutraceuticals, have gained prominence in the food industry [3].

Matcha has gained significant consumer popularity in recent years. Bamboo leaves offer advantages such as low cost and ease of harvesting, making bamboo leaf powder a promising alternative to matcha, with potential applications in foods such as cakes, ice cream, and chocolate [4]. Fixation is a critical step in the production of bamboo leaf powder and is also widely applied in tea processing, where it serves as a core procedure in primary manufacturing. It plays a decisive role in determining the color, aroma, and taste of tea, being indispensable for maintaining quality stability and shaping distinctive characteristics [5]. Steaming is effective in rapidly inactivating enzymes and preserving color, and is commonly applied in the processing of matcha and certain green teas [6]. Baking, which simultaneously dries the leaves and enhances aroma formation, is also a mainstream fixation method in green tea production [7,8]. In contrast, blanching is simpler to perform and more cost-effective, and it can also promote the dissolution of water-soluble bitter and astringent compounds, thereby improving the overall flavor of the product [9]. These high-temperature treatments inactivate enzymes, inhibit oxidative reactions, reduce chlorophyll degradation by endogenous enzymes, preserve color, and enhance aroma development [10,11]. Color is a key sensory indicator of plant material quality, with changes directly linked to chlorophyll stability and enzymatic browning. Zhang [12] demonstrated that fixation methods significantly impact chlorophyll retention, as well as polyphenol oxidase (PPO) and peroxidase (POD) activities, thereby influencing the color of mulberry leaves. The retention and transformation of volatile organic compounds (VOCs) are also critical in evaluating the fixation techniques. Han et al. [13] revealed that fixation methods greatly affect the formation of volatile compounds through variations in temperature, duration, and heating techniques, thus shaping distinctive tea aroma profiles. Jiao et al. [14] investigated the impact of different fixation techniques on orange black tea, demonstrating significant effects on its appearance, aroma, and taste characteristics. However, studies on the effects of the fixation process on the color and flavor characteristics of bamboo leaf powder remain limited. Some studies focus only on identifying the flavor compounds of bamboo leaves. For instance, Shen et al. [15] analyzed and characterized the major odor-active compounds in Phyllostachys edulis leaves, identifying 13 key aroma-active compounds such as 3-methyl-1-butanol, (E)-2-hexenal, and ethyl hexanoate. Wei et al. [16] identified a total of 142 volatile compounds from the leaves of seven bamboo species, including *Dendrocalamus latiflorus Munro* and *Dendrocalamus membranaceus Munro*. Ten compounds, including hexanal, (Z)-2-hexenal, and methyl heptanone, were commonly found across all seven bamboo species. Wang et al. [17] characterized the volatile compounds in eight bamboo species, including *Pleioblastus amarus* and *Pleioblastus maculatus*, revealing that (Z)-3-hexen-1-ol, (E)-2-hexen-1-ol, 1-hexanol, (E, E)-2,4-hexadienal, and limonene were among the most abundant. Their results also demonstrated considerable variation in aroma profiles among different bamboo species.

Therefore, in this study, the effects of three types of fixation methods, such as steaming, baking and blanching on the color of bamboo leaf powder were investigated by testing the color parameters, chlorophyll content, and POD and PPO activities. The optimal conditions for different fixation methods were achieved, and their effects on the volatile compounds and aroma characteristics of bamboo leaf powder were studied using headspace solid-phase microextraction combined with gas chromatography–mass spectrometry (HS-SPME-GC-MS), gas chromatography–olfactometry (HS-SPME-GC-O), and relative odor activity value (ROAV) analysis. These findings provide a comprehensive characterization of color and flavor development during the fixation of bamboo leaves and will be helpful for optimizing bamboo leaf powder production and its application in the food industry.

## 2. Materials and Methods

### 2.1. Materials

The 2-methyl-3-heptanone standard was supplied by J&K Chemical Co. Ltd. (Shanghai, China). Sodium dihydrogen phosphate (NaH_2_PO_4_), dibasic sodium phosphate (Na_2_HPO_4_), guaiacol, catechol, and propanone were obtained from Sinopharm Chemical Reagent Co., Ltd. (Shanghai, China). Anhydrous ethanol was acquired from Taitan Scientific Co., Ltd. (Shanghai, China). The purity of all standards was above 99%. Sinopharm Chemical Reagent Co., Ltd. (Shanghai, China) was the source of the auxiliary reagents and solvents utilized in this work.

### 2.2. Fixation of Bamboo Leaves and Powder Preparation

The leaves of bamboo (*Bambusa emeiensis*) were harvested in Ya’an (Sichuan, China) in October 2024 and provided by Senlong Biotechnology Co., Ltd. (Ya’an, China). Fresh bamboo leaves (FBLs) were vacuum-packed directly after collection without further processing and stored at 4 °C in the dark. For the untreated group, FBL were naturally dried in a cool, dark environment for 10 h. For the steamed bamboo leaves, FBL were heated in a forced-air oven at 160 °C with 100% humidity for 30, 60, 120, or 180 s. For the baked bamboo leaves, FBL were baked in the same forced-air oven at 160 °C with 0% humidity for 30, 60, 120, or 180 s. For the blanched bamboo leaves, FBL were immersed in boiling water for 15, 30, 60, or 120 s, immediately cooled in ice water to prevent further heating, and then naturally dried in a cool, dark place for 10 h. All bamboo leaf samples had a moisture content of 6.2 ± 0.5%. The untreated, steamed, baked, and blanched bamboo leaf samples were ground into powder using a grinder until they passed through an 80-mesh sieve to produce untreated bamboo leaf powder (UBL), steamed bamboo leaf powder (SBL), baked bamboo leaf powder (BBL), and blanched bamboo leaf powder (BCBL). The bamboo leaf powder samples were vacuum-packed and stored at 4 °C for subsequent experiments.

### 2.3. Determination of Color Value

Color measurement was performed according to the method described by Qin et al. [18], with slight modifications. The bamboo leaf powder samples were placed at room temperature in the dark for 6 h, after which the L*, a*, and b* values were measured using a colorimeter (WB-2000IXA, Tongde Venture Technology Co., Ltd., Beijing, China), previously calibrated with a standard white plate. Measurements were taken at three uniformly selected points from representative portions of bamboo leaf powder. The total color difference (ΔE) was calculated using Equation (1).

(1) $$\Delta E=\sqrt{{\left(L_{0}^{*}-L^{*}\right)}^{2}+{\left(a_{0}^{*}-a^{*}\right)}^{2}+{\left(b_{0}^{*}{-b}^{*}\right)}^{2}}$$

where L_0_*, a_0_*, and b_0_* represent the color values of the FBL, and L*, a*, and b* represent the color values of the bamboo leaf powder after different fixation treatments. The parameter a* indicates the red-green degree, b* indicates the yellow-blue degree, and ΔE represents the total color difference after treatments. A ΔE < 1.0 is considered imperceptible, a ΔE of 1.0–2.0 is perceptible only to trained observers, and a ΔE > 2.0 indicates a clear color difference [19].

### 2.4. Chlorophyll Content Determination

The determination of chlorophyll content was performed based on the literature [20] with slight modifications. A 0.5 g sample of bamboo leaf powder was mixed with 10 mL of extraction solvent (ethanol: acetone = 1:1, *v*/*v*) in a conical flask, sealed with parafilm, and extracted for 5 h in the dark. After extraction, the liquid was filtered to obtain the test solution. The extraction solvent was used as a blank to calibrate the spectrophotometer, and absorbance was measured at 645 nm and 663 nm. Total chlorophyll content (ω) was calculated using Equation (2).

ω = (8.05 × A_1_ + 20.29 × A_2_) × V ⁄ (1000 × m), (2)

where ω is the total chlorophyll content of bamboo leaf powder (mg/g); A_1_ is the absorbance of the test solution at 663 nm; A_2_ is the absorbance at 645 nm; V is the total volume of the test solution (mL); m is the total mass of the bamboo leaf powder sample (g).

### 2.5. Determination of POD and PPO Activities

A 0.5 g bamboo leaf sample was weighed, cut into small pieces, and ground in a pre-chilled mortar with 10 mL of 0.1 mol/L phosphate buffer (pH 6.0) in an ice bath to form a homogenate. The homogenate was centrifuged at 8000 rpm for 15 min. The pellet was re-extracted twice with the same buffer, and the supernatants were combined to obtain the crude enzyme extract, and then stored at 4 °C for analysis.

The POD activity assay was adapted from the reference [21] with slight modifications. A 0.05 mL aliquot of the crude enzyme extract was mixed with 1.0 mL of 0.1 mol/L guaiacol solution. Using the phosphate buffer as a blank, absorbance was measured at 470 nm before and 3 min after the reaction.

The PPO activity assay followed the method described in a previous study [22] with slight modifications. A 0.2 mL aliquot of the crude enzyme extract was mixed with 1.0 mL of 0.1 mol/L catechol solution. Using the phosphate buffer as a blank, absorbance was measured at 525 nm before and 3 min after the reaction.

The POD and PPO activity (U) of the bamboo leaf sample was calculated using Equation (3).

U = (∆A × V_T_) ⁄ (0.01 × m × t × V_S_), (3)

where enzyme activity (U) is defined as the amount of enzyme required to produce an absorbance change of 0.01 per minute per gram of sample, expressed in U/(g·min). The parameters are as follows: ΔA is the change in absorbance during the reaction time; V_T_ is the total volume of the crude enzyme extract (mL); m is the mass of the bamboo leaf sample (g); t is the reaction time (min); vs. is the volume of the crude enzyme extract used in the assay (mL). The enzyme activity of FBL was defined as 100%, and results for other samples were expressed as relative activity.

### 2.6. HS-SPME-GC-MS Analysis of VOCs

The extraction of VOCs was performed according to the method described by Hu et al. [23] with slight modifications. A 1.0 g sample of bamboo leaf powder was weighed accurately into a 20 mL headspace vial, and 1 μL of 2-methyl-3-heptanone (118.2 mg/L in methanol) was added as an internal standard. A pre-conditioned SPME fiber (50/30 μm DVB/CAR/PDMS) was exposed to the headspace at 45 °C for 30 min to extract volatile compounds. After extraction, the fiber was rapidly inserted into the GC injection port and held 7 min for desorption and separation.

Analysis was performed on a GC/MS-QP 2020 NX system (Shimadzu, Kyoto, Japan) equipped with an SHWAX column (30 m × 0.25 mm × 0.25 μm). The GC was operated in splitless mode with helium as the carrier gas at a flow rate of 1.0 mL/min. The injector temperature was set to 250 °C. The column temperature was initially held at 40 °C for 3 min, then increased to 230 °C at a rate of 10 °C/min, and held for 6 min. The MS was operated in electron ionization mode (positive ion, 70 eV), with the ion source temperature at 210 °C and the transfer line at 250 °C. Mass spectra were acquired in full-scan mode over a range of 33–400 amu. Volatile compounds were identified using the NIST 17 database.

### 2.7. Calculation of ROAV

The contribution of VOCs to the aroma of bamboo leaf samples depends on both their concentration and odor threshold. Their overall impact on the overall aroma can be evaluated using the ROAV. VOCs with ROAV > 1 are generally considered to have significant aromatic activity, with higher values indicating greater contributions to the overall flavor [17]. The ROAVs for volatile compounds in bamboo leaf samples were calculated using Equations (4) and (5).

ROAV_i_ = OAV_i_ ⁄ (OAV_max_ × 100), (4)

OAV_i_ = C_i_ ⁄ T_i_, (5)

where OAV_i_ is the odor activity value of the compound, OAV_max_ is the highest odor activity value of the VOCs; C_i_ and T_i_ represent the relative content of compounds and odor threshold, respectively.

### 2.8. HS-SPME-GC-O Analysis of VOCs

GC-O analysis was conducted following the method described by Geng et al. [24], with minor adjustments. The analysis utilized a Nexis GC 2030 system (Shimadzu, Kyoto, Japan) fitted with an odor detection port (ODE-2030, Shimadzu, Kyoto, Japan) to identify aroma-active compounds. Following chromatographic separation, the volatile components were evenly split (1:1) between the flame ionization detector and the GC-O sniffing port, with the transfer line temperature of 200 °C. The chromatographic column and temperature ramping protocol for the GC-O experiment mirrored those employed in the GC-MS analysis detailed in Section 2.7. A team of three trained sensory panelists performed the GC-O assessments. Employing a 4-point intensity scale (1 = weak, 2 = moderate, 3 = strong, 4 = very strong) to characterize the flavor profiles and assess the aroma intensities of the samples.

### 2.9. Sensory Evaluation

Quantitative descriptive analysis (QDA) was conducted following the standards of ISO (2016) [25] and ISO (2023) [26], with reference to the methodology of Xu et al. [27]. Twelve participants (six male, six female) were selected from students and faculty at Jiangnan University (Wuxi, China) based on their performance in screening assessments to form a sensory evaluation panel. Panelists underwent two months of training, consisting of three sessions per week on theoretical knowledge and olfactory exercises, to accurately evaluate aroma attribute intensities. Through panel discussions, eight flavor attributes highly relevant to bamboo leaf powder were selected: grass, lotus leaf-like, seaweed-like, woody (bark-like), cooked, fruity, raw (mowing-like), and roasted. The panel evaluated the intensity of each flavor attribute in bamboo leaf powder using a 5-point scale (0 = none to 5 = very strong, in 0.5-point increments). Each panelist evaluated each sample twice to ensure consistency. The sensory evaluation results were visualized using a sensory radar map.

### 2.10. Statistical Analysis

All experiments were performed in triplicate, with results reported as mean ± standard deviation. Statistical significance was determined by one-way analysis of variance (ANOVA) using IBM SPSS Statistics 23 (IBM, Armonk, NY, USA), with a significance level of *p* < 0.05 considered statistically significant. Figures were generated using Origin 2024 (OriginLab Corporation, Northampton, MA, USA). All data are expressed on a dry weight basis.

## 3. Results

### 3.1. Effect of Fixation on POD and PPO Activities in Bamboo Leaf Powder

Table 1 presents the POD and PPO activities of bamboo leaves subjected to different fixation methods and durations. UBL exhibited only a slight decrease compared with FBL, consistent with the findings of Li et al. [28] that natural storage provides limited enzymatic inhibition. POD activity decreased to less than 1% of that in FBL after steaming or baking for 120 s and blanching for 30 s, confirming the strong inhibitory effects of these treatments. Blanching for 15 s reduced POD activity by 85.25%, highlighting its high efficiency. PPO activity also declined with longer baking or blanching durations, reaching 31.48% and 17.02% of FBL after 180 s and 120 s, respectively. Blanching showed the strongest PPO inactivation, superior to steaming and baking. Steaming caused only a minor reduction, likely because moist heat did not sufficiently alter PPO structure, whereas dry heat (baking) and direct heat transfer (blanching) promoted more effective enzyme inactivation [29]. Fixation reduced POD activity more markedly than PPO, consistent with reports indicating higher PPO thermal stability in food matrices [30,31]. Feng et al. [32] demonstrated that sustained POD and PPO activities during tea storage promote continuous polyphenol oxidation, resulting in color deterioration and reduced sensory quality. Therefore, high-temperature fixation effectively inactivates POD and PPO, reducing discoloration and flavor loss.

### 3.2. Effects of Fixation on Chlorophyll Content and Color of Bamboo Leaf Powder

The changes in chlorophyll content and color values of bamboo leaf powder subjected to different fixation methods and durations are presented in Table 1. The chlorophyll content in FBL was 1.90 ± 0.03 mg/g, which decreased to 0.90 ± 0.04 mg/g in UBL after drying and grinding. This reduction was mainly attributed to degradation by endogenous enzymes and accelerated chlorophyll breakdown during processing [33,34]. With prolonged fixation, chlorophyll content first increased and then decreased. At shorter fixation durations, chlorophyll levels remained similar to those in UBL, likely because the treatment was insufficient to fully inactivate enzymes, allowing residual activity to continue degrading chlorophyll. At longer durations, chlorophyll peaked under steaming for 60 s, baking for 60 s, and blanching for 30 s, corresponding to 57.98%, 48.40%, and 80.85% of FBL, respectively. Under these conditions, partial enzyme activity and thermal damage coexisted, but the balance favored chlorophyll preservation. Among the three methods, blanching for 30 s gave the highest retention (1.55 ± 0.15 mg/g), significantly higher than that of UBL, indicating greater effectiveness than steaming or baking in preventing chlorophyll degradation. With further extension of fixation time, chlorophyll content decreased again, as prolonged heating promoted demetallation reactions despite reduced enzymatic activity [35]. Thus, chlorophyll retention depended on the interaction of temperature, duration, and moisture during fixation.

Fixation also had a significant influence on the L, a*, and b* color parameters. The L* of FBL was 49.9 ± 0.67, with no significant difference compared to UBL. Steaming for 120 s increased L* to 50.68 ± 0.24, likely due to cell wall disruption and release of intracellular components [36]. With increasing fixation duration, greenness (a*) improved, reaching its minimum at 120 s of steaming, 60 s of baking, and 30 s of blanching (−1.37 ± 0.09, −1.44 ± 0.05, −1.62 ± 0.07), all significantly lower than UBL (−1.06 ± 0.13). This reflected the high enzyme activity in UBL, which continuously promoted color deterioration, while fixation reduced enzyme activity and delayed further color loss. Chlorophyll peaks at baking 60 s and blanching 30 s mirrored a* and b* trends, confirming that optimal fixation preserved greenness. Prolonged fixation reduced greenness, increased yellowness, and caused greater deviation from FBL, indicating that long high-temperature exposure degraded green pigments and produced yellowing.

ΔE represents the color difference, with FBL as the reference. ΔE increased with fixation time, reaching 3.90 ± 0.11 after steaming for 180 s, confirming that excessive fixation promotes deterioration. The minimum ΔE values for steaming, baking, and blanching occurred at 30 s, 60 s, and 30 s, respectively, consistent with the optimal a* values for baking and blanching. Blanching for 30 s gave the lowest ΔE (0.66 ± 0.02), significantly below UBL (1.22 ± 0.24), suggesting that blanching was particularly effective in preserving the green color of bamboo leaf powder.

Based on the a* value, which directly and specifically reflects the degree of green color retention [37], the optimal fixation conditions were identified as steaming for 120 s, baking for 60 s, and blanching for 30 s. Under these conditions, the a* values were significantly lower than those at other steaming durations, and both baking and blanching markedly reduced chlorophyll loss (*p* < 0.05). These fixation parameters effectively inactivated enzymes while preserving the bright green color of the product and preventing color deterioration. Notably, when comparing the optimal conditions across fixation methods, blanching for 30 s showed a clear advantage in color preservation, consistent with the findings of Fu [38].

### 3.3. Effect of Fixation on the Aromatic Profiles of Bamboo Leaf Powder

**Figure 1:**
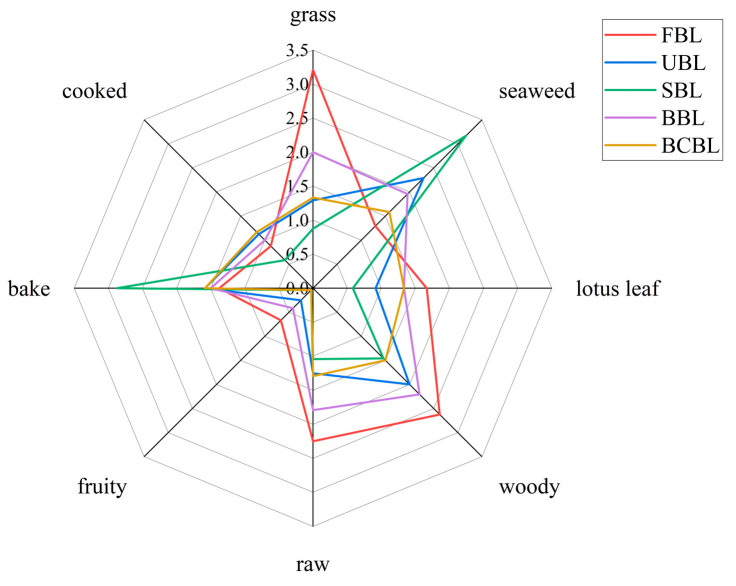
QDA radar map of bamboo leaf powder samples processed with different fixation methods. FBLs, fresh bamboo leaves; UBL, untreated bamboo leaf powder; SBL, steamed bamboo leaf powder; BBL, baked bamboo leaf powder; BCBL, blanched bamboo leaf powder.

The Quantitative Descriptive Analysis (QDA) radar chart for FBL, UBL, SBL, BBL, and BCBL is presented in Figure 1. Distinct flavor profiles were observed among the five samples, which can be explained by the different fixation methods that altered both the composition and abundance of volatile compounds, thereby shaping their aroma characteristics [39].

FBL scored higher in grass, raw, lotus leaf, and fruity notes because it underwent no thermal processing, retaining volatile compounds such as hexanal that contribute to bamboo leaf aroma. It also showed woody notes, likely from lignin-related precursors that remained unaltered in the absence of processing. In UBL, seaweed and cooked notes increased, probably from new compounds formed during dehydration and pulverization. Other odor intensities declined, especially grass, which dropped sharply from 3.21 to 1.29, indicating significant flavor loss during natural storage. This was likely due to enzymatic degradation of volatiles, while the absence of heat limited precursor transformation. Steaming enhanced seaweed-like and roasted notes, likely from intensified Maillard reactions. Both BBL and BCBL showed relatively balanced profiles, but BCBL exhibited weaker characteristic attributes, with diminished grass and woody aromas typical of FBL and reduced seaweed-like flavor compared with UBL. Although blanching involves brief heat exposure, it neither enhances key aroma notes nor effectively preserves native flavor compounds. In contrast, BBL retained grass, woody, raw, and fruity nuances more effectively, suggesting that baking better preserves the characteristic aroma compounds of bamboo leaf powder.

### 3.4. Effect of Fixation on the Volatile Compound Composition of Bamboo Leaf Powder

**Figure 2:**
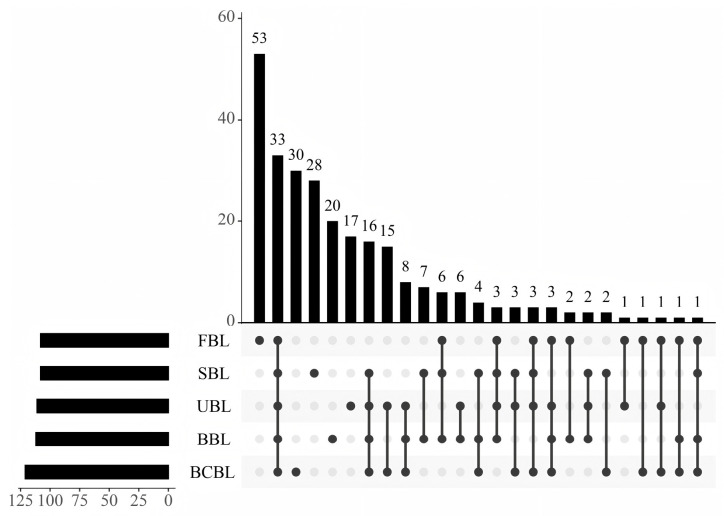
UpSet plot of volatile compounds in five bamboo leaf powder samples based on GC-MS data. FBLs, fresh bamboo leaves; UBL, untreated bamboo leaf powder; SBL, steamed bamboo leaf powder; BBL, baked bamboo leaf powder; BCBL, blanched bamboo leaf powder.

**Figure 3:**
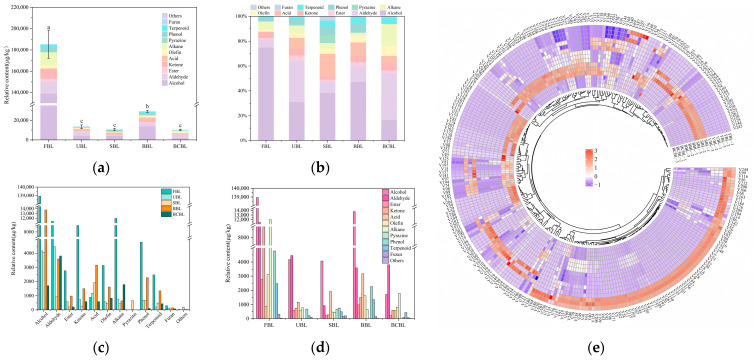
(**a**) Total content stacked column, (**b**) percentage stacked column, (**c**,**d**) classification plot, and (**e**) heatmaps of volatile compounds on the basis of GC-MS data in five bamboo leaf powder samples. In the heatmap, the numbers correspond to the compound numbers in Table A1. Different lowercase letters mean significant differences in content (*p* < 0.05). FBLs, fresh bamboo leaves; UBL, untreated bamboo leaf powder; SBL, steamed bamboo leaf powder; BBL, baked bamboo leaf powder; BCBL, blanched bamboo leaf powder.

A total of 266 VOCs were detected in bamboo leaf samples by GC-MS, with 107 in FBL, 111 in UBL, 108 in SBL, 112 in BBL, and 121 in BCBL (Appendix A, Table A1). The upset plot in Figure 2 shows that 33 volatile compounds were shared among the five bamboo leaf powders, while 53 were unique to FBL. These VOCs were classified into 12 categories: alcohols, aldehydes, esters, ketones, acids, alkanes, alkenes, pyrazines, phenols, terpenes, furans, and others. Figure 3a presents the variations and relative proportions of VOC categories across different bamboo leaf powder samples. Overall, VOC content varied significantly among samples (*p* < 0.05), with FBL having the highest total VOC content at 185.34 mg/kg, exceeding that of treated leaves. The high VOC level in FBL resulted from the absence of drying, fixation, and grinding. VOC content in UBL was 7.26% of FBL, indicating substantial losses during drying and grinding. After fixation, drying, and grinding, VOCs declined sharply to 8.53–15.67% of FBL, mainly due to reductions in alcohols, aldehydes, and ketones. These changes likely resulted from high-temperature conditions during fixation, which degraded thermally unstable VOCs, particularly alcohols, aldehydes, and ketones, through oxidation, hydrolysis, or thermal degradation reactions [40], while subsequent drying and grinding further contributed to VOC loss through volatilization and oxidative degradation [41]. SBL and BCBL retained 78.91% and 75.54% of UBL VOCs, respectively, with no significant difference in total content but clear variations in VOC species. In contrast, BBL had significantly higher VOC levels than UBL and the other two fixation treatments, increasing by 115.99% relative to UBL. This may result from stronger enzymatic reactions in UBL and the moist heat conditions of steaming and blanching, which accelerate decomposition or oxidation of flavor compounds. Moreover, the dry heat environment of baking may have intensified Maillard reactions, forming compounds such as ketones and furans, consistent with the findings of Hu [42].

Figure 3b–d present the proportions of volatile compounds across bamboo leaf powder samples and the comparative distributions of different categories. Alcohols were the most abundant VOCs in FBL, accounting for 74.97%, and played a key role in its flavor. The predominant alcohols, 1-hexanol, 3-methyl-butanol, (E)-3-hexen-1-ol, and (Z)-2-penten-1-ol, are common in fruits and vegetables and mainly originate from enzymatic fatty acid oxidation pathways [43]. Alcohols made up 31.00% of total VOCs in UBL, but only 3.00% of FBL, indicating substantial losses during drying and grinding. After steaming, the alcohol content remained largely unchanged, whereas in BCBL it was 41.00% of UBL. In contrast, baking increased alcohols by 229.92%, likely due to extensive hydrolysis of bound aroma precursors during thermal processing [44], enhancing compounds such as linalool, benzyl alcohol, phenylethyl alcohol, and 2-methoxy-4-vinylphenol. Aldehydes were the second most abundant class in FBL, accounting for 6.30% of VOCs despite only nine species being detected. In UBL, aldehydes were most abundant, with 21 species accounting for 33.08% of total VOCs (39.19% of the FBL level). Natural drying likely caused partial aldehyde degradation, while milling promoted more complete release or transformation. After fixation, aldehyde contents decreased in all treatments. Among fixed samples, BCBL retained the highest diversity and abundance (85.43% of UBL), whereas SBL had the lowest (20.82% of UBL). Hexanal, common in plants with a fresh grass odor, decreased by 58.54% after drying and grinding, but was better retained in BCBL (32.92% of FBL, 79.40% of UBL). Compared to UBL, BCBL showed increased octanal, nonanal, and decanal (149.15%, 164.52%, and 119.60%, respectively), consistent with Tang et al. [45]. This likely reflects enhanced lipid oxidation under high temperature, contributing to a slightly higher cooked aroma in BCBL. New compounds, such as (E, E)-2,4-heptadienal and (E)-2-nonenal, appeared in treated powders, though their formation pathways remain unclear.

Esters, ketones, olefins, alkanes, phenols, terpenoids, and furans were most abundant in FBL, but all experienced substantial losses after processing. In UBL, except for acids, which increased, esters, ketones, olefins, alkanes, phenols, terpenoids, and furans decreased by 69.75–93.57%, indicating inevitable flavor compound losses during drying and grinding. Terpenoid content in UBL was very low (8.69% of FBL) but increased significantly after fixation, ranging from 102.34% to 527.92%, with new compounds such as (Z)-3,7-dimethyl-1,3,6-octatriene and α-farnesene. Acids, phenols, and furans decreased after blanching, but increased after steaming and baking, particularly in BBL, where acids reached 270.49% of UBL. Esters, ketones, olefins, and alkanes were lowest in SBL, suggesting that steaming caused the most pronounced flavor loss. Notably, SBL contained unique pyrazines (668.54 μg/kg), absent in other samples. Methylpyrazine (215.4 μg/kg), primarily formed via Maillard reactions [46], likely contributed to the enhanced roasted aroma in SBL.

To visually distinguish FBL, UBL, and the three treated bamboo leaf powder samples (SBL, BBL, BCBL), a heatmap was generated using cluster analysis based on VOC content (Figure 3e), with a color scale from low (purple) to high (red). The heatmap clearly illustrates significant changes in VOC distribution after fixation treatments.

### 3.5. Screening of Aroma-Active Compounds in Bamboo Leaf Samples Using ROAVs

**Figure 4:**
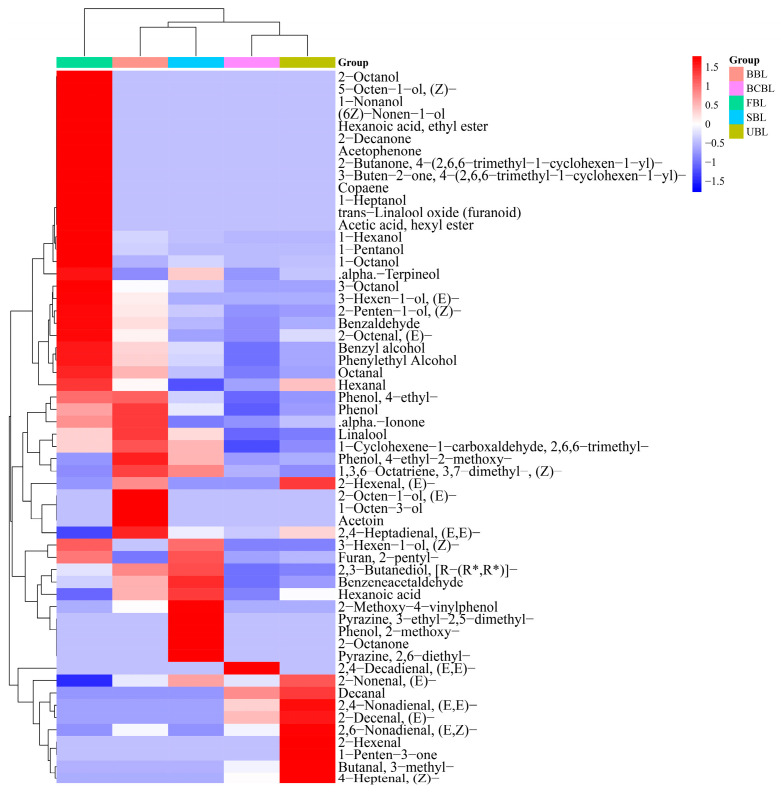
Heatmap of aroma compounds in bamboo leaf powder samples. FBLs, fresh bamboo leaves; UBL, untreated bamboo leaf powder; SBL, steamed bamboo leaf powder; BBL, baked bamboo leaf powder; BCBL, blanched bamboo leaf powder.

The aroma characteristics of bamboo leaf powder result from the combined effects of various volatile compounds. However, the overall aroma profile does not necessarily correlate directly with their concentrations. Instead, it is essential to consider their odor thresholds to evaluate their contribution to the overall aroma [47]. VOCs with an ROAV > 1 are typically considered key contributors to characteristic aromas. In this study, nonanal was assigned an ROAV of 100, and its odor threshold and description were used as references for other compounds. The ROAVs for various bamboo leaf samples are presented in Table 2, and the heatmap of aroma compounds is shown in Figure 4.

A total of 60 VOCs with ROAV > 1 were identified in the bamboo leaf samples, including 21 alcohols, 18 aldehydes, 8 ketones, 5 phenols, 2 esters, 2 terpenes, 2 pyrazines, 1 acid, and 1 furan, all significantly contributing to the basic aroma profile. Although alcohols were abundant, their high odor thresholds limited their contribution, while aldehydes with lower thresholds dominated the aroma profile. In FBL, 37 VOCs with ROAV > 1 were identified, such as β-ionone, α-ionone, hexanol, and linalool, consistent with Shen et al. [15]. These compounds, associated with green, sweet, fresh, fusel, woody, and floral notes, contributed to the high green and woody sensory scores for FBL (Figure 1). Among the 28 compounds with ROAV > 1 in UBL, 19 were also detected in FBL, indicating that bamboo leaves retained part of their fundamental aroma characteristics after drying and grinding. Additionally, several new compounds, including (E, Z)-2,6-nonadienal, (E)-2-nonenal, and (E)-2-decenal, were detected in UBL, contributing to its sensory profile.

Seven VOCs with ROAV > 1 were common to all five bamboo leaf samples: linalool (40.41–613.61), hexanal (6.09–35.96), octanal (15.00–31.12), nonanal (100), (E)-2-octenal (18.07–115.78), 2,6,6-trimethyl-1-cyclohexene-1-carboxaldehyde (2.18–13.61), and phenylacetaldehyde (3.97–48.67). These compounds mainly contribute fresh, green, and sweet aromas, consistent with the characteristic scent of bamboo leaves, and play essential roles in both pre- and post-fixation samples. As fundamental flavor components, their quantitative changes directly reflect the enhancement or loss of characteristic flavors during fixation.

Among them, five compounds had their lowest ROAVs in BCBL, while three peaked in FBL and two in BBL, respectively. This indicates that BCBL experienced the greatest flavor loss during fixation, whereas BBL maintained better flavor preservation, consistent with the results in Figure 1.

### 3.6. Effects of Fixation on the Key Aroma Compounds in Bamboo Leaf Powder

**Figure 5:**
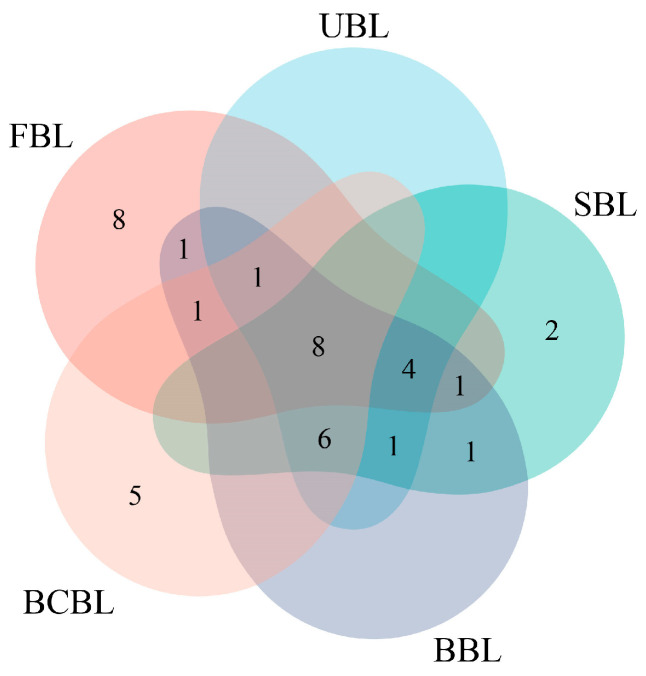
Venn diagram of volatile compounds in five bamboo leaf powder samples based on GC-O data. FBLs, fresh bamboo leaves; UBL, untreated bamboo leaf powder; SBL, steamed bamboo leaf powder; BBL, baked bamboo leaf powder; BCBL, blanched bamboo leaf powder.

GC-O combines GC-MS with human olfactory detection to identify aroma-active compounds in complex mixtures [49]. Volatile compounds detected by GC-O are generally considered key aroma-active components [50]. GC-O analysis of bamboo leaf samples processed with different fixation methods identified 39 volatile compounds: 13 alcohols, 15 aldehydes, 5 ketones, 1 ester, 5 phenols, and 1 terpene (Table 3). Among these, 33 matched the ROAV > 1 compounds listed in Table 2, while six others had ROAV < 1 or were undetected: heptanal, 2,4-nonadienal, α-ethylidene-benzeneacetaldehyde, 6-methyl-5-hepten-2-one, 1-octen-3-one, and estragole. As shown in Figure 5, eight key aroma compounds were common to all five bamboo leaf samples. They were hexanal (grass), (E)-2-octenal (fresh, cucumber, green), (E)-2-hexenal (grass, fresh, green), (Z)-3-hexen-1-ol (fresh, green, grass, foliage), 1-hexanol (fruity, sweet, rice), 3-octanol (herbal, spicy, minty), α-ionone (sweet, floral, violet), and 6-methyl-5-hepten-2-one (fruity, sweet, rice). These compounds form the core aroma framework of bamboo leaves.

GC-O analysis identified 24 key aroma compounds in FBL, consistent with previous findings on moso bamboo [15]. The most potent were estragole (spice, foul, 3.33), linalool (floral, sweet, fresh, 3.00), 2,6,6-trimethyl-1-cyclohexene-1-carboxaldehyde (herbal, clean, fruity, 3.00), phenol (foul, 3.00), 6-methyl-5-hepten-2-one (fruity, sweet, rice, 2.67), hexanal (fresh, green, grass, foliage, 2.67), ethyl hexanoate (sweet, fruity, 2.67), and 1-pentanol (oil, sweet, balsamic, 2.67). Estragole is synthesized from phenylalanine via the phenylpropanoid pathway and has a sweet, anise-like, slightly herbal aroma [51]. Linalool, a widespread terpene alcohol, substantially contributes to tea aroma [52]. 2,6,6-trimethyl-1-cyclohexene-1-carboxaldehyde exhibits intense citrus, woody, and floral notes and, as a carotenoid oxidative degradation product, forms mainly through photo-oxidation and enzymatic degradation [53]. Its concentration increases with processing temperature, aligning with the observed ROAV elevation in SBL and BBL.

UBL contained 20 key flavor compounds, 13 of which were also detected in FBL, indicating that UBL partially retains the fundamental flavor profile of bamboo leaves. Notably, (E, E)-2,4-heptadienal was detected in UBL and all three fixation samples but was absent in FBL, consistent with GC-MS results, suggesting it formed during drying and grinding. SBL contained 23 key compounds, 11 of which were shared with FBL. In BBL, 24 key compounds were identified, including 13 shared with FBL, indicating BBL had higher overall flavor abundance and better retention of characteristic compounds. SBL and BBL shared 21 key flavor compounds, and both contained the same unique compound, 4-ethyl-2-methoxyphenol, absent in the other samples, consistent with the ROAV results. This compound, mainly from lignin degradation or microbial fermentation, exhibits spicy and smoky notes [54]. BCBL contained 19 key compounds, the fewest among the samples, indicating a weaker aroma, consistent with the sensory profile in Figure 1. Only 10 compounds were shared with FBL, suggesting blanching caused the greatest loss of characteristic aroma. However, blanching retained certain key compounds better than steaming and baking, as evidenced by higher hexanal intensity in BCBL. Neither SBL nor BBL had unique key compounds. In contrast, BCBL contained five—heptanal, 1-octen-3-one, decanal, (E)-2-nonenal, and 2,4-nonadienal—highlighting the distinctiveness of blanching, which promoted new aroma formation and endowed BCBL with unique flavor attributes.

It has been shown that a compound’s contribution to overall flavor depends on interactions within the surrounding matrix. Synergistic or additive interactions between compounds with similar structures or aromas can enhance specific odors, which may explain the detection of previously undetected compounds by GC-O. Conversely, masking effects between structurally different compounds may reduce or eliminate certain perceptions [55]. This helps explain why many compounds with ROAV > 1 were not detected by GC-O. While ROAV and GC-O results were not fully identical, several overlaps were observed. For example, 1-pentanol, 3-methyl-1-butanol, heptanol, (Z)-2-penten-1-ol, acetophenone, and copaene were significant contributors in FBL by both methods. Linalool, benzyl alcohol, phenylethyl alcohol, and benzaldehyde showed low ROAVs in BCBL and were undetected by GC-O, whereas guaiacol was identified only in SBL, in agreement with ROAV results. These results demonstrate both consistency and complementarity between GC-O and ROAV [56], thereby validating our combined approach for identifying key flavor compounds in bamboo leaves.

In addition, the results showed that blanching and baking fixation each exhibited distinct advantages in color and flavor. For synergistic optimization, future studies could consider applying freeze-drying or low-temperature drying after blanching, combined with cryogenic grinding, to reduce the loss of heat-sensitive compounds. Another strategy is to extract natural flavor essences from BBL and reintroduce them into color-preserved BCBL, thus achieving a better balance between color and flavor.

## 4. Conclusions

This study established optimal fixation conditions for bamboo leaves by evaluating color parameters, chlorophyll content, and enzyme inactivation. The effects of fixation methods on aroma quality were investigated by GC-MS and GC-O. Steaming for 120 s, baking for 60 s, or blanching for 30 s effectively preserved the color of bamboo leaf powder. A total of 266 volatile compounds were identified, including 60 identified by ROAV and 39 by GC–O. Core contributors such as hexanal, nonanal, linalool, (Z)-3-hexen-1-ol, 1-hexanol, α-ionone, and β-ionone contributed to fundamental green, fresh, foliage, and floral notes. Different fixation methods produced distinct flavor profiles, with steaming generating pyrazines, thereby altering the aroma composition. Blanching was most effective for color preservation, while baking was more effective in retaining flavor compounds. Thus, blanching may be considered the preferred method for color stability, although further optimization of drying and grinding is needed to maximize flavor retention. Incorporating natural bamboo leaf extracts during fixation could further enhance aroma quality. As the sensory impacts of fixation are not fully understood, future studies should include sensory evaluation for deeper insights. In addition, research on the innovation of bamboo leaf powder processing techniques and its applications in food products remains limited, providing important directions for future research. These findings clarify the chemical basis of bamboo leaf aroma and elucidate the impact of processing on product quality, providing a foundation for producing color-stable, flavor-rich products.

## Figures and Tables

**Table 1:** Effects of different fixation methods and durations on chlorophyll content, enzyme activity, L*, a*, b*, and color difference in bamboo leaf powder.

Samples	Chlorophyll Content mg/g	PPO Relative Activity	POD Relative Activity	L*	a*	b*	ΔE
FBL	1.90 ± 0.03 ^A^	100% ^A^	100% ^A^	49.90 ± 0.67 ^BC^	−2.04 ± 0.25 ^G^	9.31 ± 0.26 ^E^	0.00 ± 0.00 ^H^
UBL	0.90 ± 0.04 ^D^	97.38% ± 7.44% ^ABC^	97.30% ± 1.20% ^B^	50.29 ± 0.67 ^AB^	−1.06 ± 0.13 ^CD^	9.65 ± 0.15 ^D^	1.22 ± 0.24 ^EF^
SBL/30 s	0.81 ± 0.04 ^DEb^	89.82% ± 8.13% ^BCa^	46.3% ± 0.82% ^Ea^	49.96 ± 0.41 ^BCb^	−1.05 ± 0.07 ^CDb^	9.66 ± 0.24 ^Dc^	1.12 ± 0.04 ^Fd^
SBL/60 s	1.06 ± 0.11 ^Ca^	85.09% ± 6.13% ^CDa^	20.48% ± 0.15% ^Fb^	48.40 ± 0.23 ^EFc^	−1.17 ± 0.05 ^Dc^	10.34 ± 0.44 ^Bb^	2.06 ± 0.08 ^Dc^
SBL/120 s	0.82 ± 0.05 ^DEb^	86.79% ± 0.9% ^BCDa^	5.19% ± 0.12% ^Hc^	50.68 ± 0.24 ^Aa^	−1.37 ± 0.09 ^Eb^	11.77 ± 0.19 ^Aa^	2.67 ± 0.18 ^Cb^
SBL/180 s	0.73 ± 0.03 ^Eb^	85.70% ± 10.43% ^BCDa^	3.54% ± 0.64% ^Id^	47.07 ± 0.24 ^Gd^	0.54 ± 0.09 ^Aa^	10.00 ± 0.21 ^Cbc^	3.90 ± 0.11 ^Aa^
BBL/30 s	0.81 ± 0.02 ^DEb^	94.82% ± 2.73% ^ABa^	83.08% ± 1.03% ^Ca^	48.84 ± 0.12 ^DEa^	−0.99 ± 0.15 ^Cb^	9.17 ± 0.19 ^EFb^	1.51 ± 0.15 ^Eb^
BBL/60 s	0.93 ± 0.08 ^CDa^	73.66% ± 4.13% ^Eb^	61.29% ± 0.76% ^Db^	48.91 ± 0.20 ^DEa^	−1.44 ± 0.05 ^Ec^	9.28 ± 0.09 ^Eb^	1.16 ± 0.15 ^Fb^
BBL/120 s	0.50 ± 0.03 ^Fc^	51.91% ± 3.18% ^Gc^	0.79% ± 0.33% ^Jc^	47.15 ± 0.17 ^Gc^	−1.05 ± 0.09 ^CDb^	10.36 ± 0.10 ^Ba^	3.11 ± 0.12 ^Ba^
BBL/180 s	0.59 ± 0.04 ^Fc^	31.48% ± 2.41% ^Hd^	0.45% ± 0.13% ^Jc^	47.14 ± 0.64 ^Gc^	−0.44 ± 0.06 ^Ba^	10.51 ± 0.14 ^Ba^	3.43 ± 0.42 ^Ba^
BCBL/15 s	0.88 ± 0.07 ^Db^	77.65% ± 3.98% ^DEa^	13.75% ± 0.00% ^Ga^	49.47 ± 0.48 ^CDa^	−1.06 ± 0.09 ^CDb^	9.06 ± 0.11 ^EFbc^	1.17 ± 0.13 ^Fb^
BCBL/30 s	1.55 ± 0.15 ^Ba^	63.82% ± 2.08% ^Fb^	0.36% ± 0.04% ^Jb^	49.60 ± 0.16 ^Ca^	−1.62 ± 0.07 ^Fc^	9.68 ± 0.09 ^Da^	0.66 ± 0.02 ^Gc^
BCBL/60 s	0.93 ± 0.09 ^CDb^	50.20% ± 5.25% ^Gc^	0.26% ± 0.04% ^Jc^	49.87 ± 0.72 ^BCa^	−1.01 ± 0.17 ^CDb^	9.32 ± 0.24 ^Eb^	1.20 ± 0.20 ^EFb^
BCBL/120 s	0.82 ± 0.06 ^DEb^	17.02% ± 6.37% ^Id^	0.21% ± 0.08% ^Jc^	48.07 ± 0.26 ^Fb^	−0.44 ± 0.06 ^Ba^	8.95 ± 0.12 ^Fc^	2.47 ± 0.16 ^Ca^

Note: Values represent the mean ± SD (*n* = 3). Different capital letters mean significant differences in fixation treatments, and different lowercase letters mean significant differences in fixation times. FBLs, fresh bamboo leaves; UBL, untreated bamboo leaf powder; SBL, steamed bamboo leaf powder; BBL, baked bamboo leaf powder; BCBL, blanched bamboo leaf powder (*p* < 0.05).

**Table 2:** The VOCs with ROAV > 1.0 in bamboo leaf samples.

NO.	CAS	Compounds	Class	Odor Description ^1^	Threshold ^2^ (μg/kg)	ROAV ^3^				
						FBL	UBL	SBL	BBL	BCBL
1	123-51-3	1-Butanol, 3-methyl-	Alcohol	fusel, oil, alcoholic, whiskey, fruity, banana	4	271.87	1.04	10.05	24.86	0.18
2	71-41-0	1-Pentanol	Alcohol	fusel, oil, sweet, balsam	120	4.34	ND	ND	0.31	ND
3	1576-95-0	2-Penten-1-ol, (Z)-	Alcohol	green, phenolic, nasturtium, ethereal, medicinal, aldehydic, cherry, narcissus, metallic, fruity	720	1.25	0.05	0.21	0.48	ND
4	111-27-3	1-Hexanol	Alcohol	ethereal, fusel, oil, fruity, alcoholic, sweet, green	5.6	374.18	3.65	14.47	33.86	ND
5	928-96-1	3-Hexen-1-ol, (Z)-	Alcohol	fresh, green, cut, grass, foliage, vegetable, herbal, oily	3.9	45.67	ND	43.87	10.47	ND
6	928-97-2	3-Hexen-1-ol, (E)-	Alcohol	green, cortex, privet, leafy, floral, petal, oily, earthy	110	9.06	ND	ND	2.83	ND
7	589-98-0	3-Octanol	Alcohol	earthy, mushroom, herbal, melon, citrus, woody, spicy, minty	18	14.96	ND	1.96	4.07	ND
8	123-96-6	2-Octanol	Alcohol	fresh, spicy, green, woody, herbal, earthy	7.8	11.39	ND	ND	ND	ND
9	3391-86-4	1-Octen-3-ol	Alcohol	mushroom, earthy, green, oily, fungal, raw, chicken	1.5	ND	ND	ND	10.80	ND
10	111-70-6	1-Heptanol	Alcohol	musty, leafy, violet, herbal, green, sweet, woody, peony	5.4	38.47	ND	ND	ND	ND
11	34995-77-2	trans-Linalool oxide (furanoid)	Alcohol	floral	60	7.07	ND	ND	ND	ND
12	24347-58-8	2,3-Butanediol, [R- (R*, R*)]-	Alcohol	buttery, sweet, fatty	95.1	2.19	0.43	5.99	4.93	0.10
13	78-70-6	Linalool	Alcohol	citrus, floral, sweet, bois, de, rose, woody, green, blueberry	1	374.12	75.76	358.39	613.61	40.41
14	111-87-5	1-Octanol	Alcohol	waxy, green, orange, aldehydic rose, mushroom	77.5	3.89	0.18	0.40	ND	0.11
15	64275-73-6	5-Octen-1-ol, (Z)-	Alcohol	green, melon, watery, watermelon, earthy, mushroom, violet, leaf, fishy, soapy	6	9.08	ND	ND	ND	ND
16	18409-17-1	2-Octen-1-ol, (E)-	Alcohol	green, citrus, vegetable, fatty	20	ND	ND	ND	1.03	ND
17	143-8-8	1-Nonanol	Alcohol	fresh, clean, fatty, floral, rose, orange, dusty, wet, oily	42	1.38	ND	ND	ND	ND
18	98-55-5	α-Terpineol	Alcohol	pine, terpene, lilac, citrus, woody, floral	4.6	103.23	17.29	51.42	ND	3.90
19	35854-86-5	(6Z)-Nonen-1-ol	Alcohol	fresh, green, melon, waxy, honeydew, cantaloupe, cucumber, clean	1	13.25	ND	ND	ND	ND
20	100-51-6	Benzyl alcohol	Alcohol	floral, rose, phenolic, balsamic	100	7.64	1.07	2.17	3.93	0.07
21	60-12-8	Phenylethyl Alcohol	Alcohol	floral, rose, dried, rose, flower, rose, water	60	7.84	1.27	2.22	4.21	0.11
22	590-86-3	Butanal, 3-methyl-	Aldehyde	ethereal, aldehydic, chocolate, peach, fatty	5	ND	1.10	ND	ND	0.22
23	66-25-1	Hexanal	Aldehyde	fresh, green, fatty, aldehydic, grass, leafy, fruity, sweaty	4.5	35.96	24.86	6.09	20.30	12.00
24	505-57-7	2-Hexenal	Aldehyde	sweet, almond, fruity, green, leafy, apple, plum, vegetable	30	ND	5.14	ND	ND	ND
25	6728-26-3	2-Hexenal, (E)-	Aldehyde	green, banana, aldehydic, fatty, cheesy	10	ND	16.00	ND	11.96	ND
26	6728-31-0	4-Heptenal, (Z)-	Aldehyde	oily, fatty, green, dairy, milky, creamy	0.2	ND	24.53	ND	ND	6.28
27	124-13-0	Octanal	Aldehyde	aldehydic, waxy, citrus, orange, peel, green, herbal, fresh, fatty	0.8	31.12	16.55	18.07	24.60	15.00
28	124-19-6	Nonanal	Aldehyde	waxy, aldehydic, rose, fresh, orris, orange, peel, fatty, peely	1.1	100.00	100.00	100.00	100.00	100.00
29	2548-87-0	2-Octenal, (E)-	Aldehyde	fresh, cucumber, fatty, green, herbal, banana, waxy, green, leaf	0.34	115.78	39.56	23.94	53.48	18.07
30	4313-3-5	2,4-Heptadienal, (E, E)-	Aldehyde	fatty, green, oily, aldehydic, vegetable, cake, cinnamon	15.4	ND	1.21	0.88	2.11	0.67
31	112-31-2	Decanal	Aldehyde	sweet, aldehydic, waxy, orange, peel, citrus, floral	3	ND	8.94	ND	ND	6.50
32	100-52-7	Benzaldehyde	Aldehyde	strong, sharp, sweet, bitter, almond, cherry	24	24.35	2.68	3.34	10.47	0.58
33	18829-56-6	2-Nonenal, (E)-	Aldehyde	fatty, green, cucumber, aldehydic, citrus	0.19	ND	156.66	125.27	76.50	76.25
34	557-48-2	2,6-Nonadienal, (E, Z)-	Aldehyde	green, fatty, dry, cucumber, violet, leaf	0.02	ND	860.91	ND	243.08	235.54
35	432-25-7	1-Cyclohexene-1-carboxaldehyde, 2,6,6-trimethyl-	Aldehyde	tropical, saffron, herbal, clean, rose, oxide, sweet, tobacco, damascone, fruity	3	9.48	4.11	10.38	13.61	2.18
36	122-78-1	Benzeneacetaldehyde	Aldehyde	green, sweet, floral, hyacinth, clover, honey, cocoa	2	15.75	9.58	48.67	32.43	3.97
37	3913-81-3	2-Decenal, (E)-	Aldehyde	waxy, fatty, earthy, green, cilantro, mushroom, aldehydic, fried, chicken, fat, tallow	0.4	ND	35.06	ND	ND	18.21
38	5910-87-2	2,4-Nonadienal, (E, E)-	Aldehyde	fatty, melon, waxy, green, violet, leaf, cucumber, tropical, fruit, chicken, fat	0.1	ND	28.06	ND	ND	12.24
39	25152-84-5	2,4-Decadienal, (E, E)-	Aldehyde	oily, cucumber, melon, citrus, pumpkin, nut, meat	0.2	ND	ND	ND	ND	14.11
40	123-66-0	Hexanoic acid, ethyl ester	Ester	sweet, fruity, pineapple, waxy, green, banana,	1	61.90	ND	ND	ND	ND
41	142-92-7	Acetic acid, hexyl ester	Ester	fruity, green, apple, banana, sweet	11	11.82	ND	ND	ND	ND
42	1629-58-9	1-Penten-3-one	Ketone	pungent, peppery, mustard, garlic, onion	0.9	ND	10.58	ND	ND	ND
43	513-86-0	Acetoin	Ketone	sweet, buttery, creamy, dairy, milky, fatty	14	ND	ND	ND	1.09	ND
44	693-54-9	2-Decanone	Ketone	orange, floral, fatty, peach	3	5.95	ND	ND	ND	ND
45	111-13-7	2-Octanone	Ketone	earthy, weedy, natural, woody, herbal	5	ND	ND	1.33	ND	ND
46	98-86-2	Acetophenone	Ketone	sweet, pungent, hawthorn, mimosa, almond, acacia, chemical	36	2.13	ND	ND	ND	ND
47	17283-81-7	2-Butanone, 4-(2,6,6-trimethyl-1-cyclohexen-1-yl)-	Ketone	earthy, woody, mahogany, orris, dry, amber	1	72.03	ND	ND	ND	ND
48	127-41-3	α-Ionone	Ketone	sweet, woody, floral, violet, orris, tropical, fruity	0.4	504.99	144.31	ND	680.09	44.45
49	14901-7-6	β-Ionone	Ketone	floral, woody, sweet, fruity, berry, tropical, beeswax	0.007	13,612.49	ND	ND	ND	ND
50	142-62-1	Hexanoic acid	Acid	sour, fatty, sweat, cheese	36	ND	0.75	1.72	1.15	0.13
51	3338-55-4	1,3,6-Octatriene, 3,7-dimethyl-, (Z)-	Triterpenoid	warm, floral, herb, flower, sweet	34	ND	ND	2.56	3.28	0.38
52	3856-25-5	Copaene	Triterpenoid	woody, spicy, honey	6	10.93	ND	ND	ND	ND
53	13360-65-1	Pyrazine, 3-ethyl-2,5-dimethyl-	Pyrazine	potato, cocoa, roasted, nutty	0.4	ND	ND	106.90	ND	ND
54	13067-27-1	Pyrazine, 2,6-diethyl-	Pyrazine	nutty, hazelnut	6	ND	ND	7.21	ND	ND
55	90-05-1	Phenol, 2-methoxy-	Phenol	phenolic, smoke, spice, vanilla, woody	1.6	ND	ND	80.87	ND	ND
56	108-95-2	Phenol	Phenol	phenolic, plastic, rubber	31	7.88	1.86	4.19	10.98	ND
57	2785-89-9	Phenol, 4-ethyl-2-methoxy-	Phenol	spicy, smoky, bacon, phenolic, clove	16	ND	0.29	2.56	4.61	0.11
58	123-7-9	Phenol, 4-ethyl-	Phenol	phenolic, castoreum, smoke, guaiacol	13	12.00	2.23	4.40	12.31	0.42
59	7786-61-0	2-Methoxy-4-vinylphenol	Phenol	sweet, spicy, clove, carnation, phenolic, peppery, smoky, woody, powdery	3	ND	ND	11.08	2.97	ND
60	3777-69-3	Furan, 2-pentyl-	Furan	fruity, green, earthy, beany, vegetable, metallic	5.8	6.24	1.48	7.07	ND	0.93

Note: ^1^ The Odor descriptions are from http://www.thegoodscentscompany.com; ^2^ The Odor threshold values of flavor compounds were obtained from the book [48]; ^3^ The “ND” in this table indicates the compounds were not detected in the sample. FBLs, fresh bamboo leaves; UBL, untreated bamboo leaf powder; SBL, steamed bamboo leaf powder; BBL, baked bamboo leaf powder; BCBL, blanched bamboo leaf powder.

**Table 3:** The aroma compounds identified in the GC-O determination of bamboo leaf samples.

NO.	CAS	Compounds	Class	Odor Description ^1^	Aroma Intensity ^2^				
					FBL	UBL	SBL	BBL	BCBL
1	123-51-3	1-Butanol, 3-methyl-	Alcohol	oil, alcoholic, fruity	1.67	ND	ND	ND	ND
2	71-41-0	1-Pentanol	Alcohol	oil, sweet, balsam	2.67	ND	ND	ND	ND
3	1576-95-0	2-Penten-1-ol, (Z)-	Alcohol	green, medicinal, mushroom	1.83	ND	ND	ND	ND
4	111-27-3	1-Hexanol	Alcohol	fruity, sweet, rice	1.33	2.33	3.67	2.33	2.50
5	928-96-1	3-Hexen-1-ol, (Z)-	Alcohol	fresh, green, grass, foliage	2.00	3.33	2.00	3.33	3.00
6	589-98-0	3-Octanol	Alcohol	herbal, spicy, minty	1.50	3.00	2.00	3.00	3.00
7	111-70-6	1-Heptanol	Alcohol	green, sweet, woody, potato	2.50	ND	ND	ND	ND
8	3391-86-4	1-Octen-3-ol	Alcohol	mushroom, earthy, herbal, woody	ND	3.00	3.00	2.00	3.50
9	78-70-6	Linalool	Alcohol	floral, sweet, fresh	3.00	3.33	3.33	3.33	ND
10	24347-58-8	2,3-Butanediol, [R- (R*, R*)]-	Alcohol	green, cucumber	ND	3.00	3.33	2.50	ND
11	98-55-5	α-Terpineol	Alcohol	woody, floral	2.00	ND	ND	3.33	3.00
12	100-51-6	Benzyl alcohol	Alcohol	floral, rose	1.67	2.67	3.00	3.33	ND
13	60-12-8	Phenylethyl Alcohol	Alcohol	floral, rose	2.00	2.33	3.00	1.50	ND
14	590-86-3	Butanal, 3-methyl-	Aldehyde	woody, grass, earthy	ND	3.00	2.00	2.00	2.50
15	66-25-1	Hexanal	Aldehyde	grass	2.67	3.00	2.50	2.00	2.67
16	6728-26-3	2-Hexenal, (E)-	Aldehyde	grass, fresh, green	1.67	3.00	3.00	2.67	2.00
17	111-71-7	Heptanal	Aldehyde	minty, fresh, green	ND	ND	ND	ND	3.50
18	124-13-0	Octanal	Aldehyde	green, fresh, lemon	ND	2.67	3.67	2.33	3.00
19	2548-87-0	2-Octenal, (E)-	Aldehyde	fresh, cucumber, green	2.50	3.00	2.50	3.00	3.00
20	124-19-6	Nonanal	Aldehyde	fresh, green, minty	ND	3.00	2.00	2.67	3.33
21	100-52-7	Benzaldehyde	Aldehyde	fresh, sweet, minty	1.67	3.00	3.00	3.00	ND
22	4313-3-5	2,4-Heptadienal, (E, E)-	Aldehyde	green, cucumber, lemon, potato	ND	3.00	3.33	3.33	3.00
23	112-31-2	Decanal	Aldehyde	sweet, floral	ND	ND	ND	ND	2.50
24	18829-56-6	2-Nonenal, (E)-	Aldehyde	green, cucumber	ND	ND	ND	ND	4.00
25	432-25-7	1-Cyclohexene-1-carboxaldehyde, 2,6,6-trimethyl-	Aldehyde	herbal, clean, fruity	3.00	ND	ND	2.67	ND
26	122-78-1	Benzeneacetaldehyde	Aldehyde	sweet, floral	2.33	3.33	ND	2.00	2.00
27	6750-03-4	2,4-Nonadienal	Aldehyde	green, cucumber	ND	ND	ND	ND	2.00
28	4411-89-6	Benzeneacetaldehyde, α-ethylidene-	Aldehyde	sweet	ND	ND	3.00	ND	ND
29	110-93-0	5-Hepten-2-one, 6-methyl-	Ketone	fruity, sweet, rice	2.67	2.67	2.33	2.00	3.00
30	4312-99-6	1-Octen-3-one	Ketone	herbal, mushroom, earthy	ND	ND	ND	ND	2.50
31	98-86-2	Acetophenone	Ketone	pungent	2.33	ND	ND	ND	ND
32	127-41-3	α-Ionone	Ketone	sweet, floral, violet	1.67	3.33	2.67	3.00	2.67
33	79-77-6	β-Ionone	Ketone	floral, violet	ND	3.67	3.50	2.50	3.50
34	123-66-0	Hexanoic acid, ethyl ester	Ester	sweet, fruity	2.67	ND	ND	ND	ND
35	3856-25-5	Copaene	Triterpenoid	honey, potato	1.50	ND	ND	ND	ND
36	140-67-0	Estragole	Phenol	spice, foul	3.33	ND	ND	ND	ND
37	90-05-1	Phenol, 2-methoxy-	Phenol	spice, woody	ND	ND	3.33	ND	ND
38	108-95-2	Phenol	Phenol	foul	3.00	ND	2.33	2.33	ND
39	2785-89-9	Phenol, 4-ethyl-2-methoxy-	Phenol	spicy, clove	ND	ND	2.33	3.00	ND

Note: ^1^ The Odor descriptions are from http://www.thegoodscentscompany.com; ^2^ The “ND” in this table indicates the compounds were not detected in the sample. FBLs, fresh bamboo leaves; UBL, untreated bamboo leaf powder; SBL, steamed bamboo leaf powder; BBL, baked bamboo leaf powder; BCBL, blanched bamboo leaf powder.

## Data Availability

The original contributions presented in this study are included in the article. Further inquiries can be directed to the corresponding author.

## Appendix A

**Table A1:** Comparative analysis of volatile organic compound profiles in bamboo leaf powder processed with different fixation methods.

NO.	Time (min)	Compounds	Class	CAS	Content (μg/kg)				
					FBL	UBL	SBL	BBL	BCBL
1	5.98	1-Propanol, 2-methyl-	Alcohol	78-83-1	2136.37 ± 659.63	ND	49.14 ± 4.50	120.05 ± 1.93	ND
2	6.306	3-Pentanol	Alcohol	584-2-1	5606.38 ± 531.21	ND	ND	ND	ND
3	6.475	(R)-[51]-2-Pentanol	Alcohol	31087-44-2	4284.21 ± 412.97	ND	ND	ND	ND
4	6.508	2-Pentanol	Alcohol	6032-29-7	ND	ND	ND	58.43 ± 11.76	ND
5	6.517	(S)-(+)-2-Pentanol	Alcohol	26184-62-3	ND	ND	40.40 ± 28.84	ND	ND
6	6.851	1-Butanol	Alcohol	71-36-3	171.17 ± 87.69	ND	ND	ND	ND
7	7.144	1-Penten-3-ol	Alcohol	616-25-1	996.73 ± 193.38	148.69 ± 6.02	92.07 ± 12.05	506.95 ± 47.03	41.09 ± 3.50
8	7.901	1-Butanol, 3-methyl-	Alcohol	123-51-3	12,768.25 ± 1393.07	29.37 ± 2.67	74.41 ± 8.01	382.70 ± 15.73	8.19 ± 2.66
9	8.57	1-Pentanol	Alcohol	71-41-0	6114.44 ± 563.15	ND	ND	142.52 ± 3.69	ND
10	9.237	1-Heptanol, 2,4-dimethyl-,	Alcohol	98982-97-9	345.70 ± 38.47	ND	ND	ND	ND
11	9.499	Cyclobutanemethanol	Alcohol	4415-82-1	ND	ND	12.73 ± 9.01	78.78 ± 6.25	ND
12	9.502	Cyclopentanol	Alcohol	96-41-3	231.86 ± 41.57	ND	ND	ND	ND
13	9.602	2-Penten-1-ol, (Z)-	Alcohol	1576-95-0	10,583.87 ± 325.07	277.72 ± 12.79	282.30 ± 33.21	1326.91 ± 46.81	ND
14	10.134	1-Hexanol	Alcohol	111-27-3	24,602.32 ± 1893.33	144.04 ± 7.32	150.09 ± 46.20	729.89 ± 16.14	ND
15	10.173	n-Pentadecanol	Alcohol	629-76-5	ND	ND	ND	ND	42.15 ± 3.64
16	10.254	3-Hexen-1-ol, (Z)-	Alcohol	928-96-1	2091.24 ± 177.69	ND	316.82 ± 179.99	157.21 ± 4.69	ND
17	10.396	1-Octanol, 2-butyl-	Alcohol	3913-2-8	ND	159.87 ± 128.15	ND	ND	561.55 ± 373.95
18	10.54	4-Penten-1-ol, 3-methyl-	Alcohol	51174-44-8	ND	283.14 ± 3.08	ND	ND	ND
19	10.548	3-Hexen-1-ol, (E)-	Alcohol	928-97-2	11,704.47 ± 934.65	ND	ND	1198.39 ± 45.55	ND
20	10.712	3-Octanol	Alcohol	589-98-0	3161.28 ± 301.18	ND	65.39 ± 8.62	281.87 ± 17.67	ND
21	10.846	2-Hexen-1-ol, (Z)-	Alcohol	928-94-9	776.90 ± 75.26	ND	ND	ND	ND
22	10.851	2-Hexen-1-ol, (E)-	Alcohol	928-95-0	ND	ND	ND	112.12 ± 4.87	ND
23	10.91	4-Hexen-1-ol, (E)-	Alcohol	928-92-7	706.36 ± 56.41	ND	ND	ND	ND
24	10.961	6-Hepten-3-ol, 4-methyl-	Alcohol	53907-71-4	1354.93 ± 101.92	ND	ND	ND	ND
25	11.062	2-Octanol	Alcohol	123-96-6	1043.08 ± 92.22	ND	ND	ND	ND
26	11.476	1-Octen-3-ol	Alcohol	3391-86-4	ND	ND	ND	62.37 ± 88.21	ND
27	11.476	1-Dodecen-3-ol	Alcohol	4048-42-4	ND	ND	ND	127.72 ± 90.74	ND
28	11.525	1-Heptanol	Alcohol	111-70-6	2439.38 ± 185.33	ND	ND	ND	ND
29	11.778	trans-Linalool oxide (furanoid)	Alcohol	34995-77-2	4983.17 ± 401.25	ND	ND	ND	ND
30	12.029	cis-Hept-4-enol	Alcohol	6191-71-5	5078.22 ± 1101.43	ND	ND	ND	ND
31	12.508	2,3-Butanediol, [R- (R*, R*)]-	Alcohol	24347-58-8	2441.91 ± 214.11	285.88 ± 26.81	1055.03 ± 233.37	1804.42 ± 64.44	111.79 ± 8.45
32	12.649	Bicyclo [4.1.0] heptan-2-ol, 3,7,7-trimethyl-, (1α,2α,3β,6α)-	Alcohol	90026-62-3	1007.54 ± 104.34	ND	ND	ND	ND
33	12.735	Linalool	Alcohol	78-70-6	4392.55 ± 369.60	533.54 ± 40.12	663.62 ± 69.98	2361.89 ± 80.64	468.23 ± 22.90
34	12.858	1-Octanol	Alcohol	111-87-5	3541.67 ± 303.73	100.28 ± 9.15	56.68 ± 6.38	ND	95.08 ± 6.49
35	12.958	2,3-Butanediol	Alcohol	513-85-9	ND	401.26 ± 140.61	ND	ND	112.06 ± 12.51
36	13.119	Ethanol, 2-(tetradecyloxy)-	Alcohol	2136-70-1	ND	ND	ND	ND	12.86 ± 14.70
37	13.127	2-Octanol, (R)-	Alcohol	5978-70-1	ND	ND	ND	20.61 ± 16.15	ND
38	13.128	Propylene Glycol	Alcohol	57-55-6	ND	ND	11.94 ± 1.88	ND	ND
39	13.427	Cyclohexanol, 2,6-dimethyl-	Alcohol	5337-72-4	387.19 ± 41.48	132.71 ± 20.94	28.49 ± 20.14	165.04 ± 9.73	89.30 ± 6.55
40	13.561	5-Octen-1-ol, (Z)-	Alcohol	64275-73-6	639.98 ± 48.26	ND	ND	ND	ND
41	13.569	2-Octen-1-ol, (E)-	Alcohol	18409-17-1	ND	ND	ND	79.42 ± 2.59	ND
42	13.897	Cyclohexanol, 5-methyl-2-(1-methylethyl)-	Alcohol	1490-4-6	ND	139.83 ± 24.91	ND	ND	101.26 ± 3.33
43	14.113	1-Nonanol	Alcohol	143-8-8	682.04 ± 53.48	ND	ND	ND	ND
44	14.171	(5-Ethylcyclopent-1-enyl) methanol	Alcohol	36431-59-1	233.87 ± 16.84	ND	ND	ND	ND
45	14.358	3-Cyclohexene-1-ethanol	Alcohol	18240-10-3	ND	ND	ND	ND	10.43 ± 0.39
46	14.396	3-Nonen-1-ol, (Z)-	Alcohol	10340-23-5	136.46 ± 10.32	ND	ND	ND	ND
47	14.4	1,9-Nonanediol	Alcohol	3937-56-2	ND	12.39 ± 0.15	ND	12.81 ± 1.03	ND
48	14.556	L-α-Terpineol	Alcohol	10482-56-1	ND	576.54 ± 73.37	ND	1387.4 ± 60.83	91.93 ± 130.00
49	14.556	α-Terpineol	Alcohol	98-55-5	5575.53 ± 161.68	560.19 ± 0.00	438.00 ± 50.22	ND	208.11 ± 147.45
50	14.76	(6Z)-Nonen-1-ol	Alcohol	35854-86-5	155.52 ± 12.61	ND	ND	ND	ND
51	14.805	2-Cyclohexene-1-methanol, 2,6,6-trimethyl-	Alcohol	6627-74-3	1238.19 ± 103.15	ND	ND	ND	ND
52	14.898	1,3-Butanediol	Alcohol	107-88-0	ND	ND	12.69 ± 8.97	ND	ND
53	15.285	(3R,6S)-2,2,6-Trimethyl-6-vinyltetrahydro-2H-pyran-3-ol	Alcohol	39028-58-5	288.30 ± 33.37	ND	ND	ND	ND
54	15.712	2,6-Octadien-1-ol, 3,7-dimethyl-, (Z)-	Alcohol	106-25-2	2259.80 ± 200.85	ND	ND	ND	ND
55	15.803	Benzenemethanol, α-methyl-	Alcohol	98-85-1	226.89 ± 22.47	31.31 ± 5.41	23.31 ± 2.67	70.17 ± 3.83	8.19 ± 1.57
56	16.278	1-Octanol, 5,7,7-trimethyl-2-(1,3,3-trimethylbutyl)-	Alcohol	36400-98-3	ND	ND	ND	ND	30.74 ± 22.17
57	16.476	Benzyl alcohol	Alcohol	100-51-6	8971.43 ± 891.56	754.23 ± 92.37	402.45 ± 52.56	1514.09 ± 76.02	85.27 ± 3.30
58	16.555	1,2-Hexanediol	Alcohol	6920-22-5	ND	ND	24.75 ± 1.08	ND	ND
59	16.863	Phenylethyl Alcohol	Alcohol	60-12-8	5521.27 ± 546.45	537.9 ± 78.74	246.63 ± 30.54	971.84 ± 45.13	74.53 ± 14.35
60	17.146	2,6,10,10-Tetramethyl-1-oxaspiro [4.5] decan-6-ol	Alcohol	77981-89-6	81.42 ± 15.08	ND	ND	ND	ND
61	17.509	1-Cyclohexene-1-propanol, α,2,6,6-tetramethyl-	Alcohol	3293-47-8	545.33 ± 46.16	ND	ND	ND	ND
62	17.522	1-Dodecanol	Alcohol	112-53-8	ND	39.70 ± 3.69	ND	ND	8.10 ± 5.77
63	17.65	β-Ethylphenethyl alcohol	Alcohol	2035-94-1	45.93 ± 5.57	ND	ND	ND	ND
64	18.233	3-Phenylpropanol	Alcohol	122-97-4	1301.93 ± 158.72	ND	ND	ND	ND
65	18.274	1,6,10-Dodecatrien-3-ol, 3,7,11-trimethyl-, (E)-	Alcohol	40716-66-3	ND	12.74 ± 3.92	4.82 ± 0.18	8.35 ± 0.93	1.88 ± 1.36
66	19.1	Cedrol	Alcohol	77-53-2	ND	ND	ND	ND	2.62 ± 1.91
67	19.966	α-Bisabolol	Alcohol	515-69-5	283.44 ± 50.87	17.31 ± 2.16	11.32 ± 1.11	41.47 ± 5.05	13.58 ± 2.96
68	20.465	2-Propen-1-ol, 3-phenyl-	Alcohol	104-54-1	22.18 ± 1.44	ND	ND	ND	ND
69	20.677	Isophytol	Alcohol	505-32-8	14.13 ± 2.72	ND	4.01 ± 3.22	28.89 ± 4.44	6.55 ± 1.55
70	21.213	3-Buten-2-ol, 3-methyl-4-(2,6,6-trimethyl-2-cyclohexen-1-yl)-	Alcohol	70172-0-8	ND	5.46 ± 0.07	ND	ND	ND
71	23.687	Phytol	Alcohol	150-86-7	ND	ND	2.61 ± 1.12	ND	0.88 ± 0.62
72	4.13	Butanal, 3-methyl-	Aldehyde	590-86-3	ND	38.58 ± 0.00	ND	ND	12.59 ± 8.92
73	5.859	Hexanal	Aldehyde	66-25-1	1899.74 ± 245.83	787.68 ± 67.18	50.71 ± 5.15	351.57 ± 13.58	625.44 ± 6.17
74	6.567	2-Pentenal, (E)-	Aldehyde	1576-87-0	ND	197.22 ± 5.93	ND	127.21 ± 13.25	105.57 ± 11.62
75	8.054	2-Hexenal	Aldehyde	505-57-7	ND	1084.96 ± 15.02	ND	ND	ND
76	8.059	2-Hexenal, (E)-	Aldehyde	6728-26-3	ND	1126.97 ± 0.00	ND	460.49 ± 27.8	ND
77	8.474	4-Heptenal, (Z)-	Alcohol	6728-31-0	ND	34.55 ± 2.37	ND	ND	14.54 ± 10.28
78	9.236	Octanal	Aldehyde	124-13-0	292.28 ± 0.00	93.22 ± 2.00	26.76 ± 2.39	75.75 ± 20.82	139.03 ± 4.87
79	10.765	Nonanal	Aldehyde	124-19-6	1291.52 ± 249.42	774.66 ± 77.68	203.68 ± 21.36	423.41 ± 25.49	1274.44 ± 71.51
80	10.976	Hexanal, 4-methyl-	Aldehyde	41065-97-8	ND	ND	ND	160.08 ± 113.3	ND
81	11.042	5-Ethylcyclopent-1-enecarboxaldehyde	Aldehyde	36431-60-4	ND	ND	ND	53.93 ± 38.15	ND
82	11.207	2-Octenal, (E)-	Aldehyde	2548-87-0	462.21 ± 36.93	94.72 ± 9.44	15.07 ± 1.21	69.99 ± 4.04	71.16 ± 3.97
83	11.646	2,4-Heptadienal, (E, E)-	Aldehyde	4313-3-5	ND	131.23 ± 38.61	25.09 ± 1.91	125.13 ± 8.29	119.13 ± 6.43
84	12.188	Decanal	Aldehyde	112-31-2	ND	188.97 ± 22.76	ND	ND	226.01 ± 21.73
85	12.406	Benzaldehyde	Aldehyde	100-52-7	6861.91 ± 761.44	452.46 ± 61.79	148.48 ± 20.71	967.35 ± 48.79	162.35 ± 8.80
86	12.622	2-Nonenal, (E)-	Aldehyde	18829-56-6	ND	209.61 ± 19.46	44.07 ± 4.42	55.94 ± 7.42	167.85 ± 16.2
87	13.246	2,6-Nonadienal, (E, Z)-	Aldehyde	557-48-2	ND	121.26 ± 14.96	ND	18.71 ± 9.74	54.58 ± 4.31
88	13.527	Undecanal	Aldehyde	112-44-7	ND	31.39 ± 4.84	ND	ND	33.36 ± 6.05
89	13.701	1-Cyclohexene-1-carboxaldehyde, 2,6,6-trimethyl-	Aldehyde	432-25-7	333.79 ± 33.93	86.84 ± 13.16	57.64 ± 6.85	157.10 ± 6.33	75.73 ± 6.10
90	13.856	Benzeneacetaldehyde	Aldehyde	122-78-1	369.76 ± 46.13	134.94 ± 11.77	180.23 ± 23.90	249.68 ± 20.32	92.09 ± 6.97
91	13.967	2-Decenal, (E)-	Aldehyde	3913-81-3	ND	98.75 ± 4.30	ND	ND	84.38 ± 17.94
92	13.998	1,3-Cyclohexadiene-1-carboxaldehyde, 2,6,6-trimethyl-	Aldehyde	116-26-7	ND	ND	96.52 ± 10.49	198.20 ± 9.08	ND
93	14.481	1-Cyclohexene-1-carboxaldehyde, 5,5-dimethyl-3-oxo-	Aldehyde	56621-35-3	136.63 ± 12.08	ND	ND	ND	ND
94	14.62	2,4-Nonadienal, (E, E)-	Aldehyde	5910-87-2	ND	19.76 ± 2.59	ND	ND	14.18 ± 1.66
95	15.193	4-Oxohex-2-enal	Aldehyde	20697-55-6	79.57 ± 4.37	39.27 ± 5.43	36.80 ± 4.83	43.48 ± 3.32	16.13 ± 1.39
96	15.237	2-Undecenal	Aldehyde	2463-77-6	ND	39.84 ± 0.40	ND	ND	ND
97	15.35	2,4-Decadienal, (E, E)-	Aldehyde	25152-84-5	ND	ND	ND	ND	32.70 ± 6.57
98	17.119	Benzeneacetaldehyde, α-ethylidene-	Aldehyde	4411-89-6	ND	ND	27.74 ± 4.62	42.37 ± 3.48	ND
99	18.227	Pentadecanal-	Aldehyde	2765-11-9	ND	7.09 ± 0.85	ND	ND	9.97 ± 7.79
100	18.605	5-Methyl-2-phenyl-2-hexenal	Aldehyde	21834-92-4	ND	ND	15.76 ± 2.23	11.4 ± 1.31	ND
101	22.385	3,5-di-tert-Butyl-4-hydroxybenzaldehyde	Aldehyde	1620-98-0	ND	ND	ND	ND	1.00 ± 0.82
102	7.639	Hexanoic acid, methyl ester	Ester	106-70-7	ND	143.89 ± 11.32	ND	ND	ND
103	8.245	2-Penten-1-ol, acetate, (Z)-	Ester	42125-10-0	ND	ND	ND	2.77 ± 0.28	ND
104	8.495	Hexanoic acid, ethyl ester	Ester	123-66-0	726.72 ± 0.00	ND	ND	ND	ND
105	8.767	4-Hexenoic acid, methyl ester	Ester	2396-79-4	ND	125.86 ± 22.74	ND	ND	ND
106	9.019	Acetic acid, hexyl ester	Ester	142-92-7	1526.70 ± 183.09	ND	ND	ND	ND
107	9.441	3-Hexenoic acid, ethyl ester	Ester	2396-83-0	ND	ND	ND	38.14 ± 1.54	ND
108	9.745	n-Caproic acid vinyl ester	Ester	3050-69-9	ND	ND	ND	16.73 ± 0.68	ND
109	11.449	Hexanoic acid, 3,7-dimethyl-6-octenyl ester	Ester	10580-25-3	834.84 ± 36.28	ND	ND	ND	ND
110	12.115	Nonanoic acid, methyl ester	Ester	1731-84-6	ND	56.46 ± 12.29	ND	22.45 ± 2.70	11.32 ± 8.17
111	13.659	Butanoic acid, 4-hydroxy-	Ester	591-81-1	ND	ND	25.96 ± 4.36	ND	ND
112	14.841	Cyclohexanol, 2,2-dimethyl-, acetate	Ester	65322-50-1	ND	ND	ND	ND	29.64 ± 1.76
113	14.89	Hexanoic acid, 1-methylethyl ester	Ester	2311-46-8	ND	ND	ND	ND	8.47 ± 6.24
114	15.475	Methyl salicylate	Ester	119-36-8	12.48 ± 15.90	ND	ND	14.63 ± 1.25	2.23 ± 1.58
115	15.845	Dodecanoic acid, methyl ester	Ester	111-82-0	37.68 ± 10.73	ND	ND	ND	ND
116	15.885	Benzoic acid, 2-hydroxy-, ethyl ester	Ester	118-61-6	103.13 ± 0.30	ND	26.92 ± 3.03	136.46 ± 11.07	ND
117	16.669	2,2,4-Trimethyl-1,3-pentanediol diisobutyrate	Ester	6846-50-0	20.38 ± 5.42	13.22 ± 2.38	ND	ND	13.27 ± 2.60
118	16.693	Benzenepropanoic acid, ethyl ester	Ester	2021-28-5	ND	ND	ND	29.69 ± 1.98	ND
119	18.05	Methyl tetradecanoate	Ester	124-10-7	ND	15.07 ± 2.50	ND	17.43 ± 2.11	ND
120	18.11	2(3H)-Furanone, dihydro-5-pentyl-	Ester	104-61-0	77.11 ± 9.58	ND	ND	ND	ND
121	18.452	Tetradecanoic acid, ethyl ester	Ester	124-6-1	ND	9.75 ± 1.05	6.86 ± 0.19	19.95 ± 0.13	8.35 ± 1.14
122	18.76	Nonanoic acid, 9-oxo-, ethyl ester	Ester	3433-16-7	ND	35.92 ± 3.29	ND	11.47 ± 1.34	21.14 ± 1.09
123	19.076	Pentadecanoic acid, methyl ester	Ester	7132-64-1	ND	23.25 ± 1.47	ND	16.47 ± 12.29	ND
124	19.271	2-Hexadecen-1-ol, 3,7,11,15-tetramethyl-, acetate, [R- [R*, R*-(E)]]-	Ester	10236-16-5	ND	ND	ND	2.57 ± 1.82	ND
125	19.561	(Z)-Ethyl pentadec-9-enoate	Ester	56219-9-1	ND	16.62 ± 3.63	9.39 ± 0.35	27.29 ± 2.28	9.80 ± 1.75
126	20.013	Benzoic acid, 2,5-dihydroxy-, ethyl ester	Ester	3943-91-7	ND	8.33 ± 1.23	4.16 ± 0.47	22.02 ± 0.99	ND
127	20.062	Hexadecanoic acid, methyl ester	Ester	112-39-0	29.14 ± 1.94	63.26 ± 19.90	16.91 ± 4.98	134.91 ± 13.94	11.96 ± 2.50
128	20.235	Ethanol, 2-(dodecyloxy)-	Ester	4536-30-5	ND	22.74 ± 9.21	ND	ND	ND
129	20.248	Hexanedioic acid, mono(2-ethylhexyl) ester	Ester	4337-65-9	ND	ND	17.93 ± 1.35	ND	ND
130	20.419	Hexadecanoic acid, ethyl ester	Ester	628-97-7	79.28 ± 10.06	72.78 ± 16.8	49.66 ± 7.53	191.24 ± 10.27	68.53 ± 12.10
131	20.842	Ethyl 9-hexadecenoate	Ester	54546-22-4	ND	3.50 ± 0.28	2.24 ± 0.26	4.74 ± 0.64	2.21 ± 0.38
132	21.089	2(4H)-Benzofuranone, 5,6,7,7a-tetrahydro-4,4,7a-trimethyl-, (R)-	Ester	17092-92-1	76.96 ± 8.67	26.8 ± 3.65	10.6 ± 0.88	19.29 ± 2.37	10.63 ± 0.87
133	22.102	9-Octadecenoic acid, methyl ester, (E)-	Ester	1937-62-8	ND	7.83 ± 1.46	2.59 ± 1.08	8.24 ± 5.83	1.74 ± 0.5
134	22.418	(E)-9-Octadecenoic acid ethyl ester	Ester	6114-18-7	ND	3.27 ± 0.11	3.92 ± 1.11	11.02 ± 1.73	2.19 ± 1.77
135	22.525	9,12-Octadecadienoic acid (Z, Z)-, methyl ester	Ester	112-63-0	ND	6.60 ± 2.51	4.42 ± 2.05	27.08 ± 3.04	2.54 ± 0.91
136	22.87	Linoleic acid ethyl ester	Ester	544-35-4	ND	ND	ND	64.94 ± 4.52	ND
137	22.875	1,2-Benzenedicarboxylic acid, bis(2-methylpropyl) ester	Ester	84-69-5	ND	30.71 ± 9.41	ND	ND	ND
138	23.177	9,12,15-Octadecatrienoic acid, methyl ester, (Z, Z, Z)-	Ester	301-0-8	ND	6.57 ± 4.27	5.43 ± 1.33	36.06 ± 5.66	ND
139	23.554	9,12,15-Octadecatrienoic acid, ethyl ester, (Z, Z, Z)-	Ester	1191-41-9	ND	4.30 ± 1.62	19.81 ± 4.69	79.04 ± 11.63	5.14 ± 3.66
140	24.622	Dibutyl phthalate	Ester	84-74-2	ND	26.87 ± 5.65	10.72 ± 2.70	29.11 ± 9.12	13.35 ± 3.33
141	4.11	3-Heptanone, 5-ethyl-4-methyl-	Ketone	27607-63-2	427.04 ± 148.89	ND	ND	ND	ND
142	4.826	1-Penten-3-one	Ketone	1629-58-9	ND	67.06 ± 0.00	ND	ND	ND
143	9.046	Acetoin	Ketone	513-86-0	ND	ND	ND	58.92 ± 0.9	ND
144	9.149	2-Decanone	Ketone	693-54-9	209.66 ± 40.73	ND	ND	ND	ND
145	9.15	Cyclooctanone	Ketone	502-49-8	ND	ND	ND	ND	8.06 ± 5.70
146	9.164	2-Octanone	Ketone	111-13-7	ND	ND	12.33 ± 5.30	ND	ND
147	9.355	3-Hepten-2-one	Ketone	1119-44-4	ND	ND	23.14 ± 2.19	ND	ND
148	9.851	6-Octen-2-one, (Z)-	Ketone	74810-53-0	ND	ND	95.39 ± 10.89	ND	11.11 ± 0.22
149	9.919	5-Hepten-2-one, 6-methyl-	Ketone	110-93-0	268.52 ± 124.89	103.45 ± 8.50	ND	68.42 ± 3.78	100.91 ± 3.15
150	12.786	1,13-Tetradecadien-3-one	Ketone	58879-40-6	ND	ND	ND	ND	1.05 ± 0.76
151	13.021	3,5-Octadien-2-one, (E, E)-	Ketone	30086-2-3	ND	41.86 ± 5.51	ND	ND	ND
152	13.038	3-Undecanone	Ketone	2216-87-7	ND	ND	ND	ND	35.28 ± 2.59
153	13.315	Isophorone	Ketone	78-59-1	1788.28 ± 141.75	75.77 ± 7.31	96.07 ± 11.07	234.98 ± 16.41	68.28 ± 4.65
154	13.987	Acetophenone	Ketone	98-86-2	902.04 ± 87.73	ND	ND	ND	ND
155	14.487	2,6,6-Trimethyl-2-cyclohexene-1,4-dione	Ketone	1125-21-9	ND	27.56 ± 5.69	ND	30.88 ± 2.77	15.18 ± 1.16
156	15.98	2-Butanone, 4-(2,6,6-trimethyl-2-cyclohexen-1-yl)-	Ketone	31499-72-6	670.96 ± 68.51	ND	2.52 ± 1.78	5.95 ± 0.84	ND
157	16.148	2-Butanone, 4-(2,6,6-trimethyl-1-cyclohexen-1-yl)-	Ketone	17283-81-7	845.75 ± 112.45	ND	ND	ND	ND
158	16.333	α-Ionone	Ketone	127-41-3	2371.64 ± 209.46	406.52 ± 21.47	ND	1047.11 ± 37.32	206.02 ± 24.15
159	16.379	5,9-Undecadien-2-one, 6,10-dimethyl-, (E)-	Ketone	3796-70-1	ND	ND	ND	ND	54.02 ± 6.97
160	17.259	β-Ionone	Ketone	14901-7-6	1118.78 ± 93.39	ND	ND	ND	ND
161	17.695	7,8-Epoxy-α-ionone	Ketone	37079-64-4	ND	35.46 ± 6.04	ND	ND	12.76 ± 2.18
162	17.899	4-(2,6,6-Trimethylcyclohexa-1,3-dienyl) but-3-en-2-one	Ketone	1203-8-3	ND	ND	20.77 ± 15.07	ND	ND
163	18.828	2,6-Di-tert-butyl-4-hydroxy-4-methylcyclohexa-2,5-dien-1-one	Ketone	10396-80-2	ND	23.6 ± 11.46	ND	ND	7.62 ± 3.05
164	19.193	2-Pentadecanone, 6,10,14-trimethyl-	Ketone	502-69-2	47.10 ± 9.83	133.61 ± 17.95	22.07 ± 0.83	43.93 ± 3.74	65.58 ± 8.02
165	20.34	1H-Pyrrole-2,5-dione, 3-ethyl-4-methyl-	Ketone	20189-42-8	20.31 ± 0.70	27.34 ± 4.25	8.31 ± 1.64	19.30 ± 3.15	5.70 ± 0.74
166	21.574	1H-Pyrrole-2,5-dione, 3-ethenyl-4-methyl-	Ketone	21494-57-5	ND	4.13 ± 0.9	ND	ND	ND
167	25.062	7,9-Di-tert-butyl-1-oxaspiro (4,5) deca-6,9-diene-2,8-dione	Ketone	82304-66-3	ND	15.15 ± 2.80	7.07 ± 1.82	11.54 ± 4.07	8.00 ± 0.74
168	4.615	Dodecane	Alkane	112-40-3	ND	ND	257.56 ± 25.57	251.09 ± 37.15	291.86 ± 411.65
169	4.616	Octane, 2,4,6-trimethyl-	Alkane	62016-37-9	ND	5.59 ± 1.2	ND	ND	ND
170	4.64	Undecane, 4,6-dimethyl-	Alkane	17312-82-2	450.14 ± 57.87	ND	ND	ND	ND
171	4.817	Nonane, 4-methyl-5-propyl-	Alkane	62185-55-1	675.10 ± 467.30	ND	ND	ND	ND
172	5.234	Dodecane, 4,6-dimethyl-	Alkane	61141-72-8	1198.39 ± 149.13	ND	ND	ND	ND
173	6.135	Heptadecane, 2,6,10,15-tetramethyl-	Alkane	54833-48-6	794.25 ± 49.13	ND	ND	ND	ND
174	6.35	Undecane	Alkane	1120-21-4	ND	ND	38.79 ± 2.84	ND	ND
175	6.365	Undecane, 3,6-dimethyl-	Alkane	17301-28-9	ND	ND	ND	ND	125.99 ± 14.83
176	6.95	5-Ethyldecane	Alkane	17302-36-2	ND	ND	2.93 ± 2.20	ND	ND
177	6.962	Tridecane, 2,5-dimethyl-	Alkane	56292-66-1	ND	37.41 ± 8.31	26.16 ± 2.47	ND	28.75 ± 2.53
178	7.46	Undecane, 3-methyl-	Alkane	1002-43-3	ND	90 ± 0.43	ND	98.86 ± 1.80	68.18 ± 5.72
179	7.46	Heptane, 2,2,3,3,5,6,6-heptamethyl-	Alkane	7225-67-4	500.19 ± 25.78	ND	ND	ND	ND
180	8.015	Hexadecane	Alkane	544-76-3	9011.18 ± 683.35	ND	ND	ND	392.50 ± 23.97
181	8.555	Undecane, 3-methylene-	Alkane	71138-64-2	ND	59.77 ± 1.40	45.32 ± 3.83	ND	31.23 ± 22.10
182	8.76	Heptadecane, 8-methyl-	Alkane	13287-23-5	1737.39 ± 122.43	ND	ND	ND	ND
183	8.889	Nonane, 5-methyl-5-propyl-	Alkane	17312-75-3	193.12 ± 84.87	ND	ND	ND	ND
184	8.89	Dodecane, 2,6,11-trimethyl-	Alkane	31295-56-4	314.87 ± 55.15	ND	ND	ND	ND
185	9.551	Heptadecane	Alkane	629-78-7	ND	143.91 ± 27.1	64.57 ± 9.45	105.42 ± 74.66	31.09 ± 22.34
186	9.706	Cyclopentane, 1,1,3-trimethyl-	Alkane	4516-69-2	ND	93.53 ± 1.81	ND	7.94 ± 1.45	94.99 ± 4.76
187	9.768	Cyclohexane, ethyl-	Alkane	1678-91-7	424.70 ± 253.46	ND	ND	ND	ND
188	10.253	Nonadecane, 9-methyl-	Alkane	13287-24-6	ND	ND	ND	ND	46.13 ± 32.96
189	10.413	Hexadecane, 2,6,10,14-tetramethyl-	Alkane	638-36-8	362.81 ± 50.8	ND	15.08 ± 1.46	27.15 ± 19.25	ND
190	10.462	Heneicosane	Alkane	629-94-7	ND	103.81 ± 58.12	ND	ND	123.51 ± 29.93
191	10.855	Cyclopentane, 1,1′-(1,4-butandiyl) bis-	Alkane	2980-70-3	ND	ND	ND	ND	37.36 ± 26.42
192	10.985	Tetradecane	Alkane	629-59-4	ND	216.64 ± 2.04	51.74 ± 37.26	ND	300.52 ± 62.27
193	11.731	Tetradecane, 3-methyl-	Alkane	18435-22-8	ND	ND	ND	ND	53.48 ± 2.46
194	11.835	Hexadecane, 2-methyl-	Alkane	1560-92-5	ND	ND	ND	ND	36.08 ± 5.68
195	12.329	Pentadecane	Alkane	629-62-9	ND	43.48 ± 6.12	ND	47.39 ± 3.18	ND
196	12.585	Triacontane	Alkane	638-68-6	66.54 ± 15.3	11.65 ± 1.24	ND	ND	ND
197	12.863	Cyclopropane, octyl-	Alkane	1472-9-9	ND	ND	ND	99.61 ± 70.96	ND
198	12.9	Tritetracontane	Alkane	7098-21-7	ND	26.41 ± 7.91	ND	ND	ND
199	13.122	Pentadecane, 1-methoxy-13-methyl-	Alkane	56196-9-9	ND	ND	ND	ND	17.52 ± 12.48
200	13.181	Pentadecane, 3-methyl-	Alkane	2882-96-4	ND	ND	ND	ND	63.42 ± 5.82
201	14.4	2-Methyltetracosane	Alkane	1560-78-7	ND	ND	ND	ND	39.65 ± 11.31
202	14.79	Hexacosane	Alkane	630-1-3	ND	49.23 ± 5.89	ND	ND	ND
203	17.178	1H-3a,7-Methanoazulene, 2,3,6,7,8,8a-hexahydro-1,4,9,9-tetramethyl-, (1α,3aα,7α,8aβ)-	Alkane	560-32-7	ND	ND	ND	ND	31.66 ± 19.55
204	10.33	Tridecane, 3-methylene-	Olefin	19780-34-8	ND	ND	ND	ND	22.81 ± 8.52
205	10.34	1-Decene, 3,3,4-trimethyl-	Olefin	49622-17-5	77.17 ± 28.39	ND	ND	ND	ND
206	10.629	2,4,4,6,6,8,8-Heptamethyl-1-nonene	Olefin	15796-4-0	ND	ND	ND	ND	13.09 ± 9.41
207	11.095	Nonane, 5-methylene-	Olefin	6795-79-5	ND	ND	ND	ND	22.96 ± 2.97
208	11.47	1-Hexene, 3,5,5-trimethyl-	Olefin	4316-65-8	ND	59.24 ± 33.84	ND	ND	77.18 ± 54.71
209	11.934	2-Hexene, 3,5,5-trimethyl-	Olefin	26456-76-8	ND	ND	ND	38.37 ± 2.16	ND
210	12.03	Cyclohexene, 3-methyl-	Olefin	591-48-0	2716.23 ± 87.14	ND	ND	ND	ND
211	13.02	Bicyclo [3.2.1] oct-2-ene, 3-(1,1-dimethylethoxy)-	Olefin	37609-41-9	ND	ND	ND	15.64 ± 19.81	ND
212	13.174	cis-α-Bergamotene	Olefin	18252-46-5	1002.97 ± 129.23	20.97 ± 3.62	30.66 ± 17.36	138.95 ± 17.11	12.16 ± 8.62
213	13.5	1,7-Octadiene	Olefin	3710-30-3	180.94 ± 2.17	ND	ND	ND	ND
214	14.62	1,4-Methano-1H-indene, octahydro-1,7a-dimethyl-4-(1-methylethenyl)-, [1S-(1α,3aβ,4α,7aβ)]-	Olefin	87064-18-4	125.07 ± 7.81	ND	ND	ND	ND
215	14.999	trans-α-Bergamotene	Olefin	13474-59-4	ND	ND	ND	ND	88.00 ± 64.01
216	17.252	Neophytadiene	Olefin	504-96-1	ND	778.38 ± 113.42	392.06 ± 49.05	1426.64 ± 87.07	561.49 ± 77.93
217	11.309	Acetic acid	Acid	64-19-7	786.62 ± 72.16	632.90 ± 58.03	1525.83 ± 181.75	2575.75 ± 304.82	371.19 ± 27.02
218	14.142	Butanoic acid, 2-methyl-	Acid	116-53-0	ND	ND	140.91 ± 15.5	200.07 ± 11.81	ND
219	14.663	Butanoic acid, 2-ethyl-, 1,2,3-propanetriyl ester	Acid	56554-54-2	ND	ND	29.61 ± 5.1	67.17 ± 11.55	29.48 ± 7.13
220	14.844	2-Hexenoic acid, 3,4,4-trimethyl-5-oxo-, (Z)-	Acid	14919-56-3	ND	54.45 ± 9.51	ND	110.23 ± 14.92	ND
221	15.437	cis-13,16-Docasadienoic acid	Acid	7370-49-2	ND	ND	ND	ND	8.19 ± 6.06
222	16.109	Hexanoic acid	Acid	142-62-1	ND	189.88 ± 18.55	114.98 ± 11.05	159.88 ± 9.98	54.09 ± 9.81
223	17.13	Undecylenic acid	Acid	112-38-9	ND	16.22 ± 3.27	ND	ND	ND
224	17.363	2-Hexenoic acid, (E)-	Acid	13419-69-7	ND	61.25 ± 10.92	32.02 ± 4.59	32.92 ± 8.30	15.13 ± 11.00
225	17.928	3-Heptenoic acid	Acid	29901-85-7	ND	32.38 ± 0.70	ND	ND	ND
226	18.339	Octanoic acid	Acid	124-7-2	59.52 ± 0.78	ND	ND	ND	ND
227	19.378	Nonanoic acid	Acid	112-5-0	69.87 ± 39.40	217.61 ± 34.9	74.06 ± 10.39	19.66 ± 18.97	107.71 ± 17.99
228	20.38	n-Decanoic acid	Acid	334-48-5	ND	ND	3.90 ± 2.86	ND	ND
229	7.815	D-Limonene	Triterpenoid	5989-27-5	ND	ND	ND	ND	2.86 ± 4.05
230	8.714	1,3,6-Octatriene, 3,7-dimethyl-, (Z)-	Triterpenoid	3338-55-4	ND	ND	161.20 ± 13.41	429.44 ± 15.03	148.57 ± 8.24
231	12.207	Copaene	Triterpenoid	3856-25-5	770.2 ± 0	ND	ND	ND	ND
232	14.309	(E)-β-Famesene	Triterpenoid	18794-84-8	309.54 ± 28.32	54.65 ± 8.29	40.50 ± 4.87	106.07 ± 4.43	33.96 ± 2.59
233	15.041	β-Bisabolene	Triterpenoid	495-61-4	951.06 ± 81.43	64.72 ± 8.38	75.00 ± 11.84	229.62 ± 6.71	64.8 ± 4.57
234	15.086	(Z)-1-Methyl-4-(6-methylhept-5-en-2-ylidene) cyclohex-1-ene	Triterpenoid	13062-0-5	394.35 ± 113.51	59.03 ± 9.64	177.04 ± 10.98	436.27 ± 20.12	78.04 ± 6.76
235	15.262	α-Farnesene	Triterpenoid	502-61-4	ND	ND	32.53 ± 2.62	75.57 ± 4.10	40.70 ± 3.84
236	15.397	Naphthalene, 1,2,3,5,6,8a-hexahydro-4,7-dimethyl-1-(1-methylethyl)-, (1S-cis)-	Triterpenoid	483-76-1	559.67 ± 43	56.09 ± 10.59	ND	71.93 ± 2.38	17.94 ± 13.17
237	15.563	Bicyclo [3.1.0] hexan-3-ol, 4-methyl-1-(1-methylethyl)-	Triterpenoid	513-23-5	ND	ND	ND	ND	47.78 ± 6.13
238	8.767	Pyrazine, methyl-	Pyrazine	109-8-0	ND	ND	215.40 ± 17.42	ND	ND
239	9.737	Pyrazine, 2,6-dimethyl-	Pyrazine	108-50-9	ND	ND	95.44 ± 10.94	ND	ND
240	10.672	Pyrazine, 2-ethyl-5-methyl-	Pyrazine	13360-64-0	ND	ND	20.99 ± 15.17	ND	ND
241	10.843	Pyrazine, 2-ethyl-3-methyl-	Pyrazine	15707-23-0	ND	ND	112.33 ± 14.32	ND	ND
242	11.429	Pyrazine, 3-ethyl-2,5-dimethyl-	Pyrazine	13360-65-1	ND	ND	79.17 ± 57.18	ND	ND
243	11.643	Pyrazine, 2,6-diethyl-	Pyrazine	13067-27-1	ND	ND	80.11 ± 45.24	ND	ND
244	11.988	Pyrazine, 2-ethenyl-6-methyl-	Pyrazine	13925-9-2	ND	ND	34.98 ± 6.74	ND	ND
245	14.383	1-(6-Methyl-2-pyrazinyl)-1-ethanone	Pyrazine	22047-26-3	ND	ND	30.11 ± 21.47	ND	ND
246	16.321	Phenol, 2-methoxy-	Phenol	90-05-1	ND	ND	239.59 ± 25.46	ND	ND
247	17.774	Phenol	Phenol	108-95-2	2868.72 ± 268.39	405.75 ± 53.38	240.47 ± 33.04	1310.53 ± 57.17	ND
248	18.108	Phenol, 4-ethyl-2-methoxy-	Phenol	2785-89-9	ND	32.39 ± 4.91	75.93 ± 10.34	284.16 ± 22.69	20.58 ± 0.75
249	19.451	Phenol, 4-ethyl-	Phenol	123-7-9	1830.97 ± 194.64	204.15 ± 25.37	105.88 ± 14.46	616.01 ± 33.17	63.54 ± 2.57
250	19.679	2-Methoxy-4-vinylphenol	Phenol	7786-61-0	ND	ND	61.56 ± 10.30	34.25 ± 5.97	ND
251	19.854	Thymol	Phenol	89-83-8	ND	ND	ND	5.57 ± 1.44	ND
252	20.767	2,4-Di-tert-butylphenol	Phenol	96-76-4	78.56 ± 7.27	43.42 ± 12.54	22.84 ± 13.01	16.73 ± 9.05	15.38 ± 3.66
253	22.143	Phenol, 3,4,5-trimethoxy-	Phenol	642-71-7	ND	ND	4.06 ± 0.75	6.71 ± 0.52	ND
254	3.777	Furan, 2-ethyl-	Furan	3208-16-0	ND	7.87 ± 1.76	ND	ND	ND
255	8.38	Furan, 2-pentyl-	Furan	3777-69-3	425.14 ± 34.84	60.46 ± 4.71	75.88 ± 54.03	ND	62.78 ± 13.59
256	11.886	5-Isopropyl-3,3-dimethyl-2-methylene-2,3-dihydrofuran	Furan	81250-44-4	ND	ND	ND	9.52 ± 6.74	ND
257	18.029	Furaneol	Furan	3658-77-3	ND	ND	45.92 ± 8.14	ND	ND
258	21.381	Benzofuran, 2,3-dihydro-	Furan	496-16-2	23.21 ± 2.07	26.75 ± 5.06	44.20 ± 7.43	141.74 ± 9.84	9.04 ± 1.09
259	13.612	1H-Pyrrole, 2-ethyl-	Others	1551-6-0	ND	ND	120.67 ± 13.03	ND	ND
260	15.398	1-Isopropyl-4,7-dimethyl-1,2,3,5,6,8a-hexahydronaphthalene	Others	16729-1-4	ND	ND	38.16 ± 27.16	ND	ND
261	16.226	3-Nonen-5-yne, 4-ethyl-	Others	74685-67-9	ND	ND	ND	ND	14.04 ± 1.86
262	16.748	10,11-Epoxycalamenene	Others	143785-42-6	27.31 ± 3.69	ND	ND	ND	ND
263	17.061	Benzyl nitrile	Others	140-29-4	ND	ND	7.01 ± 1.03	ND	ND
264	17.462	Ethanone, 1-(1H-pyrrol-2-yl)-	Others	1072-83-9	ND	ND	14.31 ± 1.93	ND	ND
265	20.296	4H-Pyran-4-one, 2,3-dihydro-3,5-dihydroxy-6-methyl-	Others	28564-83-2	ND	ND	9.55 ± 2.20	ND	ND
266	21.155	Ethosuximide	Others	77-67-8	ND	ND	0.93 ± 0.09	ND	ND

Note: Values represent the mean ± SD (*n* = 3). The “ND” in this table indicated the compounds was non-detected in the sample. FBLs, fresh bamboo leaves; UBL, untreated bamboo leaf powder; SBL, steamed bamboo leaf powder; BBL, baked bamboo leaf powder; BCBL, blanched bamboo leaf powder.
